# The Tip of the Tail Needle Affects the Rate of DNA Delivery by Bacteriophage P22

**DOI:** 10.1371/journal.pone.0070936

**Published:** 2013-08-12

**Authors:** Justin C. Leavitt, Lasha Gogokhia, Eddie B. Gilcrease, Anshul Bhardwaj, Gino Cingolani, Sherwood R. Casjens

**Affiliations:** 1 Biology Department, University of Utah, Salt Lake City, Utah, United States of America; 2 Division of Microbiology and Immunology, Department of Pathology, University of Utah School of Medicine, Salt Lake City, Utah, United States of America; 3 Department of Biochemistry and Molecular Biology, Thomas Jefferson University, Philadelphia, Pennsylvania, United States of America; Weizmann Institute of Science, Israel

## Abstract

The P22-like bacteriophages have short tails. Their virions bind to their polysaccharide receptors through six trimeric tailspike proteins that surround the tail tip. These short tails also have a trimeric needle protein that extends beyond the tailspikes from the center of the tail tip, in a position that suggests that it should make first contact with the host’s outer membrane during the infection process. The base of the needle serves as a plug that keeps the DNA in the virion, but role of the needle during adsorption and DNA injection is not well understood. Among the P22-like phages are needle types with two completely different C-terminal distal tip domains. In the phage Sf6-type needle, unlike the other P22-type needle, the distal tip folds into a “knob” with a TNF-like fold, similar to the fiber knobs of bacteriophage PRD1 and Adenovirus. The phage HS1 knob is very similar to that of Sf6, and we report here its crystal structure which, like the Sf6 knob, contains three bound L-glutamate molecules. A chimeric P22 phage with a tail needle that contains the HS1 terminal knob efficiently infects the P22 host, *Salmonella enterica*, suggesting the knob does not confer host specificity. Likewise, mutations that should abrogate the binding of L-glutamate to the needle do not appear to affect virion function, but several different other genetic changes to the tip of the needle slow down potassium release from the host during infection. These findings suggest that the needle plays a role in phage P22 DNA delivery by controlling the kinetics of DNA ejection into the host.

## Introduction

Tailed bacteriophage virions deliver DNA to susceptible cells after adsorbing to specific receptors on the surface of bacteria. In the Gram negative bacteria these receptors are surface proteins or polysaccharides. The phage virion proteins that bind to these receptors reside at the tip of the tail and usually have fibrous or elongated shapes. Considerable information is known about phage virion proteins that bind various bacterial receptors, but much less is known of the detailed mechanism of DNA release from the virion into the cell. The best understood systems are *Myoviridae* phages such as T4 and P1 with the well-known contraction of their long tails during DNA delivery [1], [2], although questions remain about exactly how this process is controlled and carried out, as well as the energetics of the process [3]. The *Myoviridae* virions insert a preassembled tube through the host membranes that functions as a conduit for DNA transit into the cytoplasm [1]. On the other hand, DNA delivery by the *Siphoviridae* (long non-contractile tails) and *Podoviridae* (short tails) is much less well understood [4], [5]. The *Podoviridae* in particular have been shown to release some virion protein molecules, called ejection proteins, along with the DNA. These proteins are required for successful DNA injection, but they have no pre-existing structure that could deliver the DNA through the membranes and periplasm [4], [6], [7]. The short-tailed phage T7 has recently been shown to rearrange its virion proteins during injection to build a structure that could serve as such a conduit, but the details of this structure and exactly how it might function remain mysterious [8], [9]. In addition, little is known about the trigger mechanism that signals the virion to release its DNA for any phage.

*Podoviridae* in the P22-like group bind their O-antigen polysaccharide primary receptors through a virion protein called the tailspike [10], [11]; six trimers of the tailspike polypeptide protrude from the sides of the base of the short tail [12]–[15]. In addition to giving these phages specificity for initial target cell recognition (*e.g.*, *Salmonella enterica* serotype Typhimurium specificity for phage P22), their tailspike proteins have a catalytic activity that hydrolyzes the polysaccharide receptor backbone. This cleavage may be important in bringing the virion close to the surface, but simple polysaccharide binding cannot be sufficient for rapid and spatially controlled DNA release from the virion [4]. Purified *S. enterica* serotype Typhimurium lipopolysaccharide (LPS, which contains the O-antigen polysaccharide) causes a rather slow P22 DNA release, suggesting that other factors could play a role in this process [16]. The short tails of the virions of P22 and its relatives are assembled from only four phage-encoded proteins. Twelve and six molecules of gp4 and gp10 proteins, respectively, form the body of its stubby tail. The six trimeric tailspikes (above) are bound to the lower sides of the tail, and a single trimer of the product of P22 gene *26* (gp26) forms a long “needle” that extends outward from the center of the base of the tail [12], [17]–[21]. This needle extends well beyond the outer radius of the tailspikes, suggesting that it should make first contact with the surface of the outer membrane during adsorption. In addition, a few copies of ejection proteins gp7, gp16 and gp20 are released from the virion during injection [13], [22]–[24], but their detailed roles remain unknown.

The N-terminal, virion-proximal end of the gp26 needle forms the exit channel plug that traps packaged DNA in the virion. In its absence DNA is packaged normally, but the particles without plugs are very unstable and DNA falls back out of the capsid, a process that starts even before cell lysis [19], [25]. In addition, gp26 is missing from wild type virions that have injected their DNA into *Salmonella* cells [24]. Thus an attractive model has been that gp26 serves as the sensor that determines when DNA should be released from the virion and then triggers its release. We have previously reported the structures of the tail needle proteins from phage P22 and its close relative phage Sf6. The trimeric needles of various P22-like phages range from about 220–320 Å long and have shafts that are only 20–30 Å wide. The long shaft domains have a three-strand coiled-coil structure with 11–16 repeats of heptad amino acid sequences, and the N-terminal 27 residues fold back on the surface of the coiled-coil in the P22 structure to form what may be the site through which the needle binds in the tail channel to form the plug. Although they are homologous in their N-terminal virion-binding domain, P22 and Sf6 have completely different domains at their C-termini; the biological reason for this is not known [26], [27], [28]. We report here the X-ray structure of the C-terminal domain of the phage HS1 needle (HS1 is an *E. coli* P22-like phage with an Sf6-like needle) which confirms the very unusual feature that this protein domain binds three L-glutamate molecules. In addition, experiments are presented that examine the function of the C-terminal needle domain.

## Results and Discussion

### Recent Evolutionary Arrival of the Sf6 Type Tail Needle Knob Domain into the P22-like Phage Group

**Figure 1:**
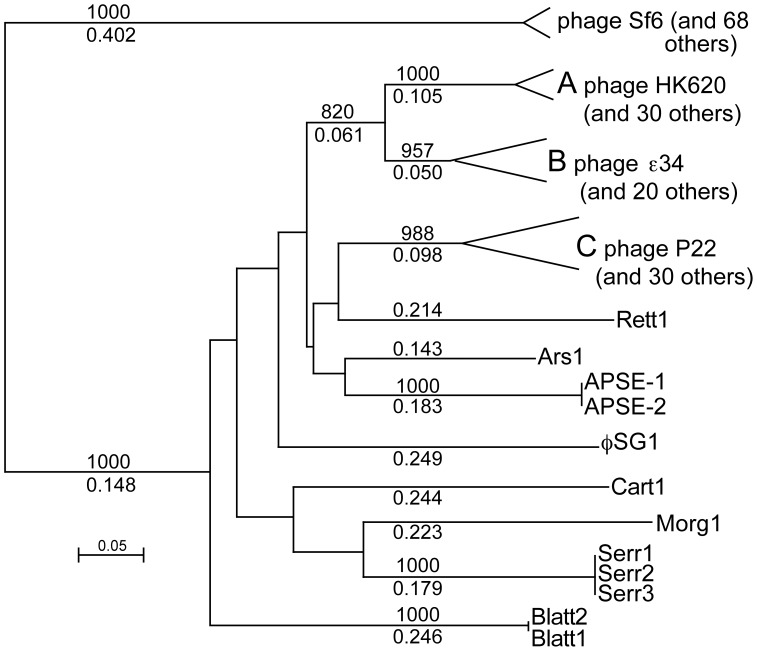
Relationships among tail needle C-terminal domains of the P22-like phages. A neighbor-joining tree (created with Clustal X2 [80]) is shown with selected branch lengths (numbers between 0. and 1) and bootstrap values out of 1000 trials (between 1 and 1000). The nodes far from the branch tips are not well-supported and are not shown. A scale in fractional difference is shown in the lower left. Branches A, B and C have many members and the splits at these branch tips show the regions within which the individual members diverge (the larger tree in figure S1 shows the placement of all the individual sequences). The “phage Sf6” branch is not related to the other branches and its inclusion here is to demonstrate this, and does not imply any phylogenetic relationship with the other branches. The branches not labeled “phage” are from P22-like prophages in the following bacterial genome sequences: Rett1, *Providencia rettgeri* DSM 1131; Ars1, *Arsenophonus nasoniae*; APSE-1/−2, *Hamiltonella defensa*; øSG1, *Sodalis glossinidius*; Cart1, *Pectobacterium carotovorum* PBR1692; Morg1, *Morganella morganii* KT; Serr1/2/3, *Serratia plymuthica* strains AS9, AS12 and AS13; Blatt1/2, *Escherichia blattae* strains DSM 4481 and 105725.

There are currently 164 genome sequences available for P22-like phages and prophages that infect 12 different species of *Enterobacteriaceae* bacteria (we make the reasonable assumption that prophages arrived at their present locations by the normal infection route). We also note that there are over 100 *E. coli* genomes that carry a small, several gene remnant of P22 that includes a homologue of the Sf6 tail needle gene; these latter genes are not exceptional and were only included in this analysis by the inclusion of the gene from *E. coli* strain EC4113 which carries such a phage genome fragment (locus_tag ECH7EC4113_3989 in defective prophage EC4113-1; our provisional prophage name). Among these 164 phage genomes, 69 of the needle genes encode Sf6 type C-terminal knob domains (*i.e.*, have sequences similar to Sf6 amino acids 140 to 282) and 95 have C-terminal domains that are related to the P22 needle tip. All of these Sf6 type knob domains are very closely related, with the most distantly related knob domains being only 4.9% different in amino acid sequence (see figure S1), and all the P22-like phages with this type of needle domain infect only bacteria in the *Escherichia* or *Shigella* genera. On the other hand, the P22-like C-terminal domains are much more diverse, with the most distant pairs being about 50% different in amino acid sequence (figures 1 and S1). Neighbor-joining tree analysis shows that there are currently eleven major branches (*i.e.*, sequence types) of the P22 type needle tip domain (figure 1). Three of these have many known members and are encoded by phages that infect *Escherichia* or *Salmonella* bacterial genera - subtype A, whose members all infect *E. coli* (typified by phage HK620 [29]), B, whose members infect *E. coli, S. enterica* or *Cronobacter sakazakii* (typified by phage epsilon 34 [30]), and C, whose members all infect *S. enterica* (typified by phage P22); in addition, still more diverse homologs are found in phages that infect other *Enterobacteriaceae* species. A possible simple explanation for lack of Sf6 tip domain diversity is that the P22 type needle tip domain is ancestral in the P22-like phage group, and it has been there long enough to allow considerable divergence to occur, while DNA encoding the Sf6 type needle knob has entered to P22-like phage population quite recently and has not had time for much divergence. This direction of transfer is supported further by the observation that there are no database matches to the P22 type needle tip domain outside the P22-like phages, but there are a number of such matches to the Sf6 type tip domain (see below). This scenario seems quite plausible because the P22-like phages are known to have undergone considerable horizontal transfer of genetic information, both within the P22-like group and with other phage types, in the genes that encode their virion assembly proteins [31].

The evolutionary source of the Sf6 type tail needle knob domain is not known; however, there are currently 21 convincing database matches of the Sf6 knob to proteins that fall outside the P22-like phage knob branch by neighbor-joining analysis (figure S2). These “outside” matches range from about 37% to 63% identical to the knobs of needle proteins present in the P22-like phages, and all are encoded by phages that are outside the P22-like group. One of these phages is an *Aeromonas* prophage, nine are somewhat phage Mu-like *Vibrio* prophages (our unpublished analysis), and the remaining eleven reside in *bona fide* phage genomes. The latter phages are all large virulent T4-like phages that infect *Enterobacteriaceae* species *E. coli, S. enterica* and *Dickeya solani* (formerly *Erwinia chrysanthami)* or *Aeromonas salmonicida.* In all of these “outside match” proteins, none of whose function is known, the homology to the Sf6 knob lies at or near the C-terminus of the protein. The phage 31, 44RR2.8 t and RB43 homologues have an N-terminal extension of up to 143 amino acids, but others have only a very short N-terminal extension in addition to the knob domain homology.

### Atomic Structure of the HS1 Knob Domain

The Sf6 tail needle knob domain has the very unusual feature that three glutamate molecules are tightly bound in the crevices between the three subunits [26]. To determine if these are unique to Sf6 or are also present in other Sf6-like tail needle knobs, we carried out structural analysis of bacteriophage HS1 tail needle knob. HS1 was identified as an apparently intact P22-like prophage in *E. coli* strain HS [31]. The HS1 tail needle (encoded by locus_tag EcHS_A0316 gene) is characterized by a virion-binding N-terminal domain 98% identical to that of Sf6, and a shaft-forming coiled-coil helical region predicted to be 210 Å long that is only 67% identical in sequence to Sf6. This helical core contains 16 heptad repeats, compared to 11 in the Sf6 and P22 needles. The C-terminus of the HS1 tail needle contains a domain (the knob) that is 99% identical to Sf6 in sequence. We cloned the HS1 prophage needle’s C-terminal knob and nine residues of its coiled-coil helical shaft fused to maltose binding protein (MBP) after polymerase chain reaction (PCR) amplification from *E. coli* strain HS DNA. The resulting hybrid protein was affinity purified, and the MBP portion removed by PreScission protease and column chromatography (details in Materials and Methods). The purified HS1 knob was crystallized, and its structure was solved by Molecular Replacement using the phage Sf6 knob domain (pdb code 3RWN) as search model, and the structure was refined to an R_work/free_ of 15.13%/15.70 at 1.1 Å resolution (Table 1 and Materials and Methods).

**Table 1:** Crystallographic data collection and refinement statistics.

Data collection statistics	
Wavelength (Å)	0.9537
Space group	p212121
Unit cell dimensions (Å)	*a* = 56.78, *b* = 87.36, *c* = 88.84
Angles (°)	α = β = γ = 90
Resolution range (Å)	20-1.1
Wilson *B*-factor (Å^2^)	8.94
Total observations	1,053,320
Unique observations	176,986
Completeness^a^ (%)	99.1 (92.8)
Redundancy^a^	6.0 (5.3)
*R*_sym_ a,b (%)	7.2 (49.9)
<I>/<σ(I)>^a^	29.96 (2.4)
**Refinement statistics**	
Number of reflections (10-1.1 Å)	176,905
*R*_work_/*R*_free_ c (%)	15.13/15.70
Number copies in asymm. unit	1
Number of water molecules	754
*B* value of model (Å^2^) chains A/B/C/waters	10.6/10.37/10.91/24.22
r.m.s. deviation from ideal bond length (Å)/angles (^°^)	0.008/1.3
Ramachandran plot (%)core/allowed/generously allowed/disallowed	92.7/7.3/0.0/0.0

a Highest resolution shell is shown in parenthesis.

b *R_sym_ = ∑_i,h_*|*I(i,h) – <I(h)>*|*/∑_i,h_*|*I(i,h)*| where *I(i,h)* and *<I(h)>* are the *i*th and mean measurement of intensity of reflection *h*.

c The *R*_free_ value was calculated using 2,000 reflections.

**Figure 2:**
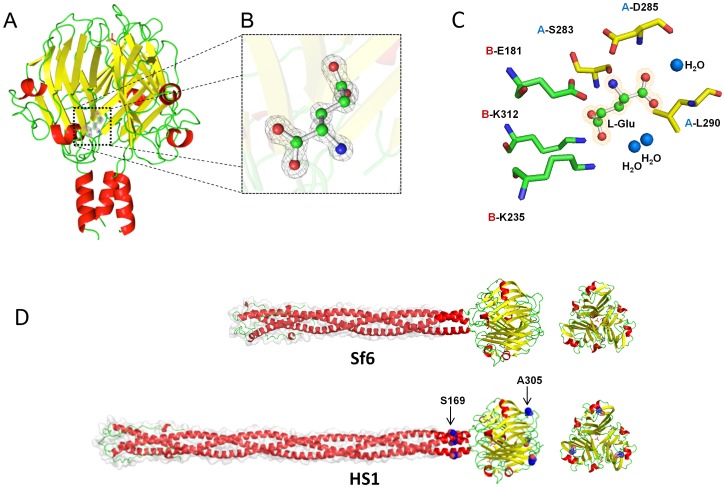
Atomic structure of the phage HS1 tail needle knob. **A**. Ribbon diagram of bacteriophage HS1 tail needle knob determined crystallographically to 1.1 Å resolution; the N-termini are at the bottom of the diagram. Helices are shown in red, ß-sheets in yellow, and random coil in green; the bound L-glutamate is shown as sticks-and-balls and phosphate is shown as a small red sticks. **B.** Magnified view of L-glutamate trapped at the HS1 needle knob dimeric protomer:protomer interface. L-glutamate (in stick-and-balls) is overlaid to the final 2Fo-Fc electron density map (gray) contoured at 1.5σ above background. **C.** Side chains (sticks) from protomer A (yellow) and protomer B (green) that interact with L-glutamate (stick-and-balls). The indicated HS1 needle amino acids correspond to Sf6 needle amino acids as follows with Sf6 residue numbers in parentheses: Glu181(146), Lys235(200), Ser283(248), Asp285(250), Leu290(255) and Lys312(277). **D.** Structural models of full length Sf6 and HS1 tail needles. The two amino acid differences (from the Sf6 needle) that lie at positions in or near the knob domain, Ser169 and Ala305 of the HS1 tail needle, are shown as blue spheres. The models were obtained by using the Robetta full-chain protein structure prediction server [81]; the N-terminal parts of the needle protein shafts whose structures are modeled from the homologous P22 tail needle have a light gray surface contour behind. In all the panels, α-helices, β-strands and loops are colored in red, yellow and green, respectively.

The globular HS1 needle knob folds into a homotrimeric TNF-like fold, very similar to that of the Sf6 knob (rmsd 0.137 Å; figure 2). The trimer is built with three identical subunits each displaying a jellyroll topology characterized by seven stacked anti-parallel ß-strands. Like the Sf6 knob [26], the HS1 tail knob structure contains a phosphate ion bound at the distal tip of the knob (figure 2A) and an L-glutamate is present in the electron density at each of the three dimeric interfaces of the homotrimer (figure 2B). The phosphate ion makes polar contacts with HS1 needle residues S266 and N268 near the 3-fold axis at the distal tip of the knob. Alteration of these residues in Sf6 knob did not affect the stability of the protein [26]. The conserved triad, S266-R267-N268, is present in all the known needle knob sequences from P22-like phages; however, although R267 (its side chain points down and is involved in intra-chain stability) is universally present in all the proteins aligned in figure S3, the S and N on either side are not conserved in proteins encoded by the non-P22-like phages, so the bound phosphate is unlikely to be a universal feature of all of these proteins. Although not surprising given the similarity of the two proteins, the presence of the bound glutamate molecules in the HS1 structure (figure 2A) is consistent with their being universally present in the P22-like needle knob structures. Remarkably, in all 21 of the non-P22-like phage knob homologues (above) amino acids that are in intimate contact with the bound L-glutamate molecules are completely conserved (Glu181, K235, S283 and D285 in the HS1 needle protein; figures 2C and S3), so it is very likely that all these proteins have bound L-glutamate. Finally, the Sf6 and HS1 tail needle knob structures have one amino acid difference within the knob domain, HS1 Ala305 (neutral hydrophilic) corresponds to Sf6 Ser270 (weak hydrophobic), and one difference in the common shaft portions whose structures are known, residue HS1 Ser169 (acidic) to Sf6 Asp134 (neutral hydrophilic) (both are indicated in Figure 2D). These differences have no effect on the overall three dimensional structure of the tail needle knob and slightly decease the negative charge of HS1 helical shaft.

### Is the C-terminal Knob of the Tail Needle Host Species-specific?

**Figure 3:**
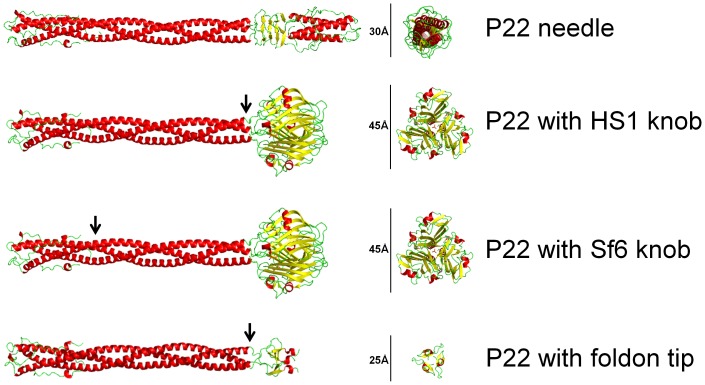
Structural models of the P22 gp26 tail needle and chimeric tail needles. **A**. Crystal structure of P22 tail needle gp26 (pdb 3C9I). **B–D**. Homology structural models of chimeric P22 needles with Sf6 knob (in phage UC-0911), HS1 knob (in phage UC-0926) and foldon tip (in phage UC-0927). Chimeric models were generated for illustration with align function of PyMol (Version 1.3, Schrodinger, LLC, San Carlos, CA), where C-terminal knob domains of Sf6 and HS1 tail needle (pdb 3RWN, 4K6B), C-terminal foldon domain from fibritin fiber of the bacteriophage T4 (pdb 1AA0) were fused downstream of P22 gp26 tail needle helical core residues 1–140 (pdb 3C9I), respectively. In all three models, arrow indicates point of fusion.

As mentioned above, the Sf6 type needle tip domain has a different fold from the P22 needle tip (figure 3). Since proteins with homology to this domain have been found only in phage that infect *E. coli, Escherichia fergusonii* and *S. flexneri* (and it has been argued that at least *E. coli* and *Shigella* are actually one species [32]), it seemed possible that this domain participates in adsorption and/or DNA delivery in a host species-specific manner. The tailspike is known to confer host primary adsorption specificity for the P22-like phages by binding to the O-antigen surface polysaccharide [31], but to test the idea that the needle protein knob might also contribute the ability to infect different hosts, we constructed two hybrid P22 *Salmonella* phages in which the distal C-terminal tip of gp26 is replaced by the Sf6 or HS1 sequence (figure 3; see Methods and Materials). These constructs were made in a P22 prophage since modifications can be made there even if the change is lethal to phage growth, and the functionality of the mutant phage can be tested by analyzing the phenotype after induction of the prophage to lytic growth. The prophage used for these tail needle modifications was P22 *sieA^–^*Δ1, *15^–^*ΔSC302::Kan^R^, *13^–^am*H101 (phage strain UC-0911), in which the three mutations allow efficient tailspike gene expression after induction, easy kanamycin selection for lysogens, and control of lysis, respectively, is capable of a normal lytic growth cycle [33]. Modifications of this prophage in *Salmonella* strain UB-1790 were made using recombineering technology (see Materials and Methods; bacterial and phage strains used are listed in Table 2).

**Table 2:** Phage and bacteria used in this study.

Bacterial strain^a^	Genotype^b^	Reference
UB-0001	(DB7000) *leuA* ^–^414, *sup°*	[62]
UB-0002	(DB7004) *leuA* ^–^414, *supE*	[62]
UB-0020	(MS1868) *leuA* ^–^414, *hsdSB* (r^–^ m^+^) Fels2^–^, *sup°*; from M.Susskind	[75]
UB-0134	*leuA^–^am*414, Fels2^–^, *cob^–^*ΔCRR299 (P22 s*ieA^–^*44, *ant^–^am*222, ΔAp68 (tpfr49 *a1^–^, 9^–^, c2^+^, mnt^+^*)); from J. Roth	[76]
UB-1732	*E. coli* HS; from J. Nataro	[77]–[80]
UB-1737	(TH2788) *fliY* ^–^5221::Tn10dTc; from K. Hughes	
UB-1790	UB-20 *galK*::TetRA-1 (P22 *sieA^–^*Δ1, *15^–^*ΔSC302::Kan^R^, *13^–^*amH101)	[33]
UB-1807	UB-20 *galK*::TetRA-1 (P22 *26*::Sf6-3::*galK*-1, *sieA^–^*Δ1, *15^–^*ΔSC302::Kan^R^, *13^–^*amH101)	
UB-1832	UB-20 (P22 *sieA^–^*Δ1, *15^–^*ΔSC302::Kan^R^, *13^–^*amH101)	
UB-1918	UB-20 (P22 *26*::Sf6-3, *sieA^–^*Δ1, *15^–^*ΔSC302::Kan^R^, *13^–^*amH101)	
UB-1919	UB-20 (P22 *26*::Sf6-3 Glu146Asp(GAC), *sieA^–^*Δ1, *15^–^*ΔSC302::Kan^R^, *13^–^*amH101)	
UB-1920	UB-20 (P22 *26*::Sf6-3 Glu146Ala(GCG), *sieA^–^*Δ1, *15^–^*ΔSC302::Kan^R^, *13^–^*amH101)	
UB-1921	UB-20 (P22 *26*::Sf6-3 Ser248Thr(ACC), *sieA^–^*Δ1, *15^–^*ΔSC302::Kan^R^, *13^–^*amH101)	
UB-1922	UB-20 (P22 *26*::Sf6-3 Ser248Ala(GCC), *sieA^–^*Δ1, *15^–^*ΔSC302::Kan^R^, *13^–^*amH101)	
UB-1924	UB-20 (P22 *26*::Sf6-3 Asp250Ala(GCT), *sieA^–^*Δ1, *15^–^*ΔSC302::Kan^R^, *13^–^*amH101)	
UB-1925	UB-20 (P22 *26*::Sf6-3 Lys200Ala(GCG), *sieA^–^*Δ1, *15^–^*ΔSC302::Kan^R^, *13^–^*amH101)	
UB-1926	UB-20 (P22 *26*::Sf6-3 Lys200Glu(GAG), *sieA^–^*Δ1, *15^–^*ΔSC302::Kan^R^, *13^–^*amH101)	
UB-1927	UB-20 (P22 *26*::Sf6-3 Glu146Ala/Asp250Ala, *sieA^–^*Δ1, *15^–^*ΔSC302::Kan^R^, *13^–^*amH101)	
UB-1928	UB-20 (P22 *26*::Sf6-3 Lys200Ala/Asp250Ala, *sieA^–^*Δ1, *15^–^*ΔSC302::Kan^R^, *13^–^*amH101)	
UB-1929	UB-20 (P22 *26*::Sf6-3 Lys200Ala/Glu146Ala, *sieA^–^*Δ1, *15^–^*ΔSC302::Kan^R^, *13^–^*amH101)	
UB-1940	UB-20 (P22 *26*::Sf6-3::TetRA-1, *sieA^–^*Δ1, *15^–^*ΔSC302::Kan^R^, *13^–^*amH101)	
UB-1941	UB-20 *galK*::TetRA-1 (P22 *26*::foldon-1, *sieA^–^*Δ1, *15^–^*ΔSC302::Kan^R^, *13^–^*amH101)	
UB-1942	UB-20 (P22 *26*::Sf6-3::TetRA-2, *sieA^–^*Δ1, *15^–^*ΔSC302::Kan^R^, *13^–^*amH101)	
UB-1943	UB-20 (P22 *26*::Sf6-3::TetRA-3, *sieA^–^*Δ1, *15^–^*ΔSC302::Kan^R^, *13^–^*amH101)	
UB-1944	UB-20 (P22 *26*::Sf6-3::TetRA-4, *sieA^–^*Δ1, *15^–^*ΔSC302::Kan^R^, *13^–^*amH101)	
UB-2078	UB-20 *galK*::TetRA-1 (P22 *26*::galK-2, *sieA^–^*Δ1, *15^–^*ΔSC302::Kan^R^, *13^–^*amH101)	
UB-2083	UB-20 *galK*::TetRA-1 (P22 *26*::HS1-1, *sieA^–^*Δ1, *15^–^*ΔSC302::Kan^R^, *13^–^*amH101)	
UB-2130	(TH18984) *fliC*5469::MudK, Δ*hin-fljA*8068::PfliC-cat, *rfbF*::TPOP; from K. Hughes	
**Phage P22 strains**		
UC-0011	P22 *c1*-7, *26^+^, 13^–^*amH101	[12]
UC-0911	P22 *26^+^, sieA^–^*Δ1, *15^–^*ΔSC302::Kan^R^, *13^–^*amH101	
UC-0918	P22 *26*::Sf6-3, *sieA^–^*Δ1, *15^–^*ΔSC302::Kan^R^, *13^–^*amH101	
UC-0926	P22 *26*::HS1-1, *sieA^–^*Δ1, *15^–^*ΔSC302::Kan^R^, *13^–^*amH101	
UC-0927	P22 *26*::foldon-1, *sieA^–^*Δ1, *15^–^*ΔSC302::Kan^R^, *13^–^*amH101	
UC-0931	P22 *26*::Sf6-3 Glu146Ala/Asp250Ala, *sieA^–^*Δ1, *15^–^*ΔSC302::Kan^R^, *13^–^*amH101	
UC-0932	P22 *26*::Sf6-3 Lys200Ala/Asp250Ala, *sieA^–^*Δ1, *15^–^*ΔSC302::Kan^R^, *13^–^*amH101	
UC-0933	P22 *26*::Sf6-3 Lys200Ala/Glu146Ala, *sieA^–^*Δ1, *15^–^*ΔSC302::Kan^R^, *13^–^*amH101	

a All strains are *S. enterica* serovar Typhimurium LT2 derivatives, except UB-1732 which is *E. coli*.

b Strain names in parentheses are the names used in the laboratory from which the strain was obtained. UB-20 in the “Genotype” column indicates derivatives of parental strain UB-0020. Mutant prophage codons are shown in square brackets; multiple mutants have the same codon changes as single mutants.

The fusion point of the above P22 and Sf6 hybrid tail needle protein was made in the coiled-coil shaft domain in a region of very high sequence similarity between the two genes, so no disruptions of trimerization heptad number or frame were made; the resulting hybrid protein should thus be very likely to fold normally into a functional needle protein. In this hybrid phage, P22 gene *26* codons 69–233 are replaced by phage Sf6 gene *9* codons 69–282, so the C-terminal knob and the C-terminal approximately two-thirds of the coiled-coil shaft have Sf6 sequence. In the HS1 hybrid needle construct, only the C-terminal knob-encoding region (codons 174 to 317 of the phage HS1 gene) neatly replaces the P22 C-terminal domain (codons 141–233) (figure 3). When the *Salmonella* strains carrying these two prophages (strains UB-1918 and UB-2083, respectively) were induced with mitomycin C, a yield of phage particles that make approximately normally sized plaques on indicator strain UB-0002 was produced in both cases that was similar to that of the isogenic strain UB-1790 whose prophage has an all-P22 tail needle (Table 3). Plating for plaques at 30°C or 37°C gave the same results. Thus, the virions of these two hybrid phages are stable and functional under laboratory conditions. We also found that both of these hybrid phages (UC-0918 and UC-0926) infect *S. enterica* serovar Typhimurium normally in liquid culture. Comparison of the proteins present in CsCl gradient purified virions with P22, Sf6 or HS1 needle tips (UC-0911, UC-0918 and UC-0926, respectively) showed identical virion proteins except that, as expected, the needle protein’s apparent molecular weight is commensurately larger when the larger Sf6 or HS1 knob domain is present (shown for the HS1 hybrid in figure S4). Since these two hybrid phages infect *Salmonella* apparently normally, we conclude that the Sf6 knob domain function does not confer species specificity to P22 in the laboratory. More formally, this shows that the P22 tip domain is not essential, and its replacement by the Sf6 needle knob does not prevent infection of *Salmonella* by P22 by adding a *Shigella*-specific component to the infection process. However, the observation that in the face of the extensive horizontal exchange among the P22-like phages [31], none of the 48 known P22-like phages that infect *Salmonella* carry the Sf6 type needle tip domain suggests there may be an evolutionary disadvantage should a *Salmonella* P22-like phage obtain a needle gene with this domain.

**Table 3:** Effects of genetic modification the C-terminal needle domain.

Phage source^a^	Lysate titer^b^	Virion infectivity^c^
UB-1790 Parent P22 phage^d^	1.7×10^10^	1.0
UB-2083 HS1-1 hybrid needle phage	7.5×10^9^	1.1
UB-1918 Sf6-3 hybrid needle phage	6.2×10^9^	2.1
UB-1919 Glu146Asp	5.5×10^9^	0.8
UB-1920 Glu146Ala	5.2×10^9^	1.7
UB-1925 Lys200Ala	1.4×10^9^	2.0
UB-1926 Lys200Glu	2.6×10^9^	1.4
UB-1921 Ser248Thr	6.3×10^9^	2.0
UB-1922 Ser248Ala	3.8×10^9^	2.1
UB-1924 Asp250Ala	2.1×10^9^	2.9
UB-1927 Glu146Ala/Asp250Ala	1.8×10^10^	1.0
UB-1928 Lys200Ala/Asp250Ala	1.4×10^9^	1.8
UB-1929 Glu146Ala/Lys200Ala	2.9×10^9^	1.3
UB-1941 Foldon	8.3×10^9^	0.13

a *Salmonella* lysogens are listed that were induced to prepare stocks of the phages in the table; the amino acid changes in the L-glutamate binding site of the needle are shown after the Sf6-hybrid needle phage strain names (UB-1919 to 1929).

b Lysogens were induced with mitomycin C and titered on *Salmonella* strain UB-0002; average results from several replicates are shown.

c Phage particles were purified through CsCl step gradients [68], and phage titers were determined on *Salmonella* strain UB-0002. Relative PFU/particle ratios were calculated by normalization to UB-1790 titer (PFU) and to the intensity of quantified coat protein bands in SDS electrophoresis gels and/or OD_280_ (particles). Several replicates were performed for each phage with similar results, and the average value is shown.

d The prophages all have the following genetic background: P22 *26*::Sf6-3, *sieA^–^*Δ1, *15^–^*ΔSC302::Kan^R^, *13^–^*amH101 (see table 2).

### Is L-glutamate Binding Essential?

In order to begin to address the biological relevance of the L-glutamate molecules present in the crystal structures of the HS1 and Sf6 needle protein knob domains, recombineering was used to engineer point mutational changes of Sf6 amino acids Glu146, Lys200, Ser248 and Asp250 (corresponding to Glu181, Lys235, Ser283, Asp285 in HS1 knob, respectively; figure 2B) that make close contact with this small molecule (above; see figure 3C in [26]). Changes were made in the Sf6 hybrid needle P22 prophage of bacterial strain UB-1918 (table 2) as follows: Glu146 to Asp or Ala, Lys200 to Glu or Ala, Ser248 to Thr or Ala, Asp250 to Ala, and combinations of the Ala changes (see details in Methods and Materials). Induction of these mutant prophages resulted in approximately normal yields of plaque-forming phage particles in all cases, including the double mutant L-glutamate binding site mutant virions (table 3). It is very likely that these changes, especially in the double mutants, would substantially lower L-glutamate binding. Therefore, the fact that all of these mutational changes do not affect virion function as measured by plaque formation suggests that L-glutamate binding by the hybrid tail needle’s distal domain is not essential under our laboratory conditions. Since the experiments here and in the previous section were performed with a hybrid needle protein in the context of P22 infection of *Salmonella*, the possibilities remain that the Sf6 knob could have a specific role in allowing phage Sf6 to infect *Shigella* and that bound L-glutamate could have an important role in a *Shigella* infection.

### The C-terminal P22 Tail Needle Domain is not Essential Under Laboratory Conditions

Because the above domain switches and the above amino acid changes did not greatly affect virion function, we wondered whether the tip domain of the gp26 needle is in fact essential for phage P22 virion function. Since removal of the C-terminal domain of the P22 needle lowers the stability of the trimer and because a translation stop at codon 54 (the P22 *26* minus *amber* H204 mutation is a C to A change at position 160 in the *26* gene) gives a *26* null phenotype when not suppressed, we deemed it prudent to replace the needle’s C-terminal domain with a “trimer nucleation domain” rather than simply C-terminally truncate the protein. The phage T4 fibritin “foldon” domain has been shown to mediate trimerization and folding of fibritin and other coiled-coil trimers when it is present at the C-terminus of these proteins [34]–[38]. Fibritin is the neck fiber protein encoded by the T4 *wac* gene. Its role in T4 is to aid in tail fiber assembly, and there is no indication that it interacts with target cells in any way. We have previously shown that fusing the foldon sequence to the C-terminus of N-terminal P22 needle protein fragments results in properly folded and very stable trimers [39]. We therefore used recombineering to replace the P22 tail needle tip domains III and IV (P22 amino acids 141–233) [27] with the 25 amino acid foldon as described in Materials and Methods to create *Salmonella* strain UB-1941 (figure 3). Induction of this prophage by mitomycin C released approximately normal numbers of progeny phages (table 3), showing that the C-terminal domains of the P22 needle are not absolutely essential in laboratory infections. This phage (UC-0927) does, however, make tiny plaques on indicator strain UB-0002, and its particle/plaque-forming unit (PFU) ratio is about 10-fold lower than the UC-0911 parent with a wild type needle (table 3), suggesting that there is a moderate (but not absolute) defect in these virions under laboratory conditions.

### The C-terminal Tail Needle Domain Affects the Rate of DNA Delivery into Cells

**Figure 4:**
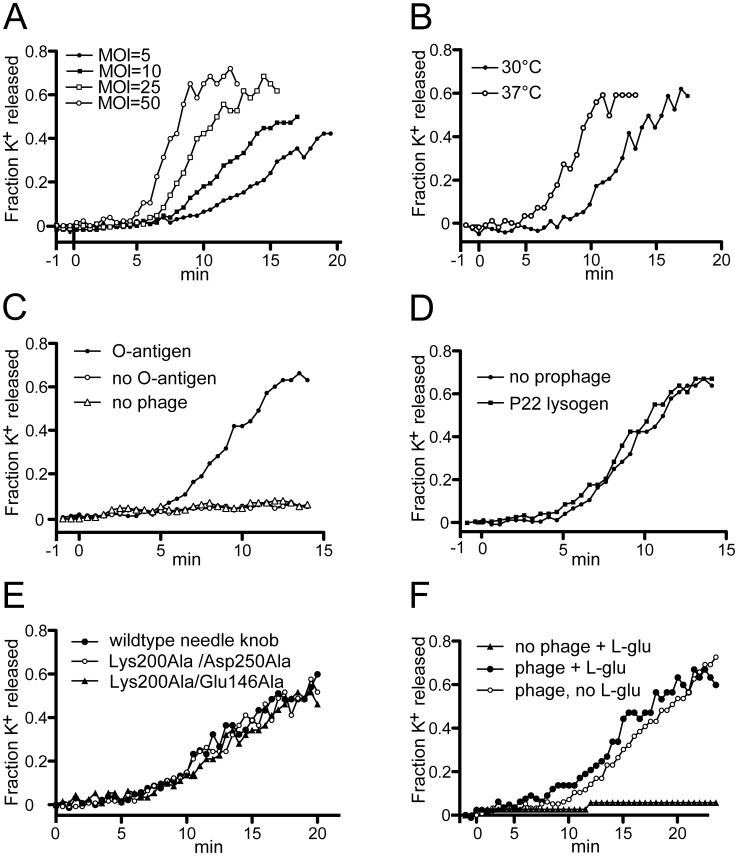
Potassium ion release by phage P22 infection. **A.** Infection of *Salmonella* strain UB-0001 by P22 clear mutant phage UC-0011 at different multiplicities of infection (MOIs). Full strain genotypes at given in Table 2 of article text. **B.** Infection of *Salmonella* UB-0001 at 30° and 37°C by phage P22 UC-0011. **C.** Infection of *Salmonella* host strains that have (UB-0001) or do not have (UB-2130) P22’s O-antigen surface polysaccharide receptor by phage P22 UC-011; potassium ion release by uninfected *Salmonella* UB-0001 is also shown for comparison. **D.** Infection of a *Salmonella* host that has no P22 prophage (UB-0001) and a host that carries a P22 prophage that expresses its repressor (*c2*) gene but is missing the *sieA* and *gtrABC* genes (UB-0134) by P22 UC-0011. **E.** Infection of *Salmonella* UB-0001 by P22 phages that carry two mutations in the tail needle knob that should abrogate L-glutamate binding (see Table 2 of article text for amino acid changes). All infections were carried out at 30°C and MOI of 10 unless otherwise indicated. **F.** Infection of *Salmonella* UB-0001 by P22 UC-0011 with and without 10 mM L-glutamate added to the medium outside of the cells. Potassium ion measurements were performed as described in Materials and Methods of article text.

Since plaque formation is not a quantitative measure of virion function, DNA might still be delivered into the cell more slowly by the P22 phages with modified tail needles (above) than phages with wild type needles. We therefore sought to measure DNA passage from the virion into the cell on a more nearly real-time scale. Entry of a number of tailed phage DNAs into the host cell cytoplasm during injection is highly correlated with an efflux of K^+^ ions out of the cell into the surrounding medium [40]–[43]. Thus, measurement of K^+^ release with an Orion Ionplus potassium electrode (see Materials and Methods) can be used as an approximate surrogate for real-time measurement of DNA entry into the cytoplasm of the cell during most tailed phage infections. P22 had been shown by Ter-Nikogosian *et al.* [44] to exhibit such ion release, and figure 4 confirms that infection by phage P22 UC-0911 causes a rapid release of K^+^ ions; however, we did not observe the dependence of K^+^ release on the presence of externally supplied Ca^++^ ions reported by those authors (data not shown and N. Cumby, personal communication). We performed the following characterizations of this system to test whether P22-induced K^+^ release correlates with its DNA delivery into susceptible cells. The kinetics and extent of K^+^ release by P22 is affected by the multiplicity of infection (MOI) (figure 4A), as might be expected from mass action considerations. All subsequent experiments were performed at the more physiologically relevant MOI of 10. P22 mediated K^+^ release is more rapid at 37°C than at 30°C (figure 4B), and we used 30°C in subsequent experiments in order to maximize resolution of any timing differences between wild type and mutant phage infections. As expected, *S. enterica* that lacks P22’s O-antigen receptor (UB-02130) shows no K^+^ release in response to added phage P22 (figure 4C). In addition, release of K^+^ is not affected by the ability or inability of the infecting phage to form a lysogen, since a clear plaque mutant (UC-0011) shows similar K^+^ release to phage that can lysogenize (UC-0911) (data not shown). Finally, P22 infection of a host that carries a P22 prophage that is missing its *sieA* and *gtrABC* genes (UB-0134) gives normal K^+^ release (figure 4D). This lysogen is defective in superinfection exclusion [45], [46] and O-antigen modification [46], [47] so DNA is injected normally, but the resident P22 prophage repressor is present in the cell and prevents expression of nearly all of the genes of the infecting P22 genome [45], [46]. Thus, expression of the vast majority of P22 genes is not required for K^+^ release. These findings are all consistent with the idea that DNA transit into the cytoplasm from adsorbed P22 virions correlates with K^+^ release by a mechanism that remains to be elucidated (see for example [48]).

Our measurements indicate that at 30°C DNA K^+^ release begins at 7 to 10 min after P22 infection and is complete by 15–20 min (figure 4); strikingly, 60–80% of cellular potassium ions are released during this time period. The lag time is affected by MOI (figure 4A) and temperature (figure 4B), as might be expected if the lag reflects the time it takes for P22 virions to bind the cell surface O-antigen.

polysaccharide and then make their way to the surface of the outer membrane (the latter probably through cleavage of the O-antigen [4]). The period during which K^+^ is released should reflect the *maximum* time it takes to transfer the DNA from the virion into the cell, and since K^+^ release is a bulk solution measurement and the infections in these experiments are not highly synchronized, DNA entry transit time for individual virions is likely significantly less than this. This transit time value of 8–13 min is sensible in terms of P22’s life cycle (reviewed in [46]), which requires that DNA circularize (and therefore have both DNA ends internalized) soon after infection begins [49]–[52]. The rate of DNA entry into the cell has not been previously measured for P22, but the above *minimum* rate of 55–95 bp/sec at 30°C. Thus the P22 value may not be very different from the approximately 160 bp/sec (about 5 min total transit time) recently measured for individual phage lambda virions at room temperature [53]; lambda is an *E. coli Siphoviridae* phage with a genome of similar size to that of P22 that also must circularize soon after infection begins. We note that DNA cell entry rates are not uniform among tailed phages, and where they have been measured entry rates range from about 3000 bp/sec for phage T4 [42] to 70–250 bp/sec for phage T7 [54]–[56] and 160 bp/sec for lambda [53]. DNA entry rate appears to be a property that is evolutionarily optimized for each phage’s molecular lifestyle.

**Figure 5:**
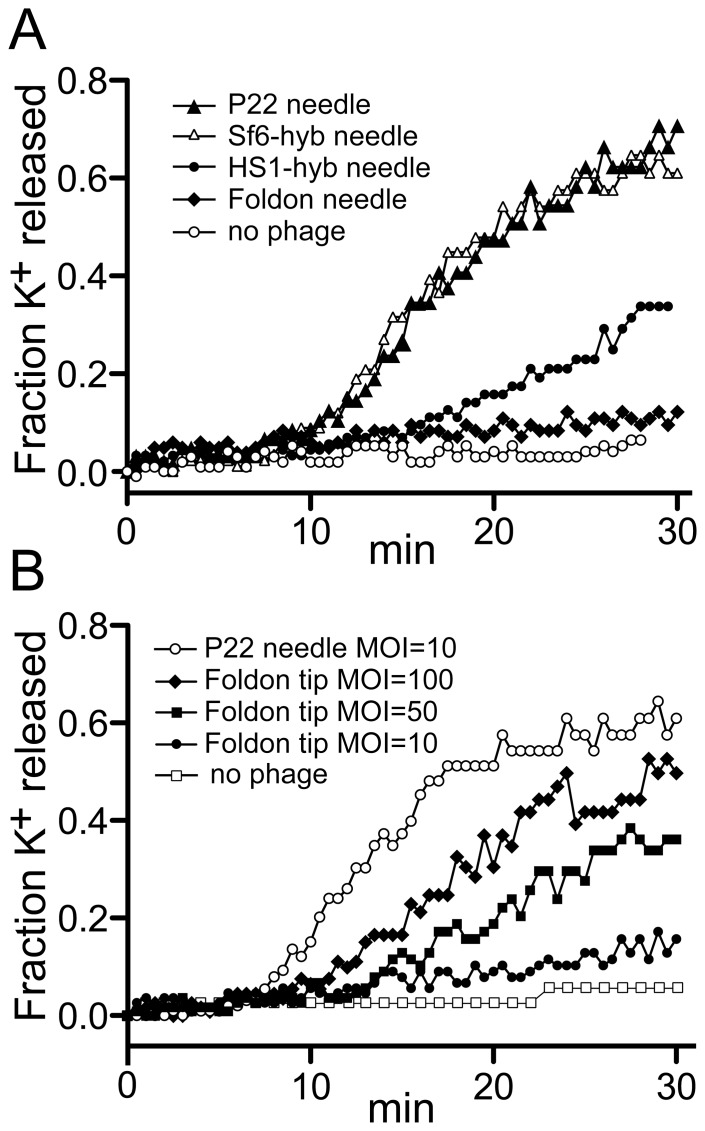
Potassium ion release by P22 phages with modified tail needles. **A.** Infection of *Salmonella* strain UB-0001 by P22 phages with altered needle proteins at MOI = 10. Potassium ion release was measured at 30°C as described in Materials and methods with a potassium electrode. *Salmonella* host strain UB-0001 was infected by the following phages: UC-0911 fully P22 tail needle (▴); UC-0918, needle has Sf6 C-terminal knob domain and part of the shaft (▵); UC-0926, needle has HS1 C-terminal knob domain (•); UC-0927, foldon replaces needle C-terminal domain (⧫); no phage infection (○). **B.** Infection of *Salmonella* strain UB-0001 by P22 phages with foldon-tipped needle at various MOIs as follows: P22 UC-0911 with fully P22 tail needle at MOI = 10 (○); P22 UC-0927 where foldon replaces needle’s C-terminal domain at MOI = 10 (•), MOI = 50 (▪) and MOI = 100 (⧫); no phage (□). The horizontal axis is time after infection.

Parallel infections by P22 with a wild type needle (UC-0911) and the isogenic phage carrying the phage HS1 C-terminal knob domain (the *26::*HS1-1 hybrid needle gene in phage UC-0926) shows significantly delayed K^+^ release kinetics for the HS1 hybrid (figure 5A). Phage UC-0926 reproducibly has a slower K^+^ “release period” which begins after a lag time that is about the same as wild type; the rate of release ranged from 40–70% of wild type in different experiments. On the other hand, P22 phage with the C-terminal tip domain and most of the needle shaft domain replaced by Sf6 sequences (UC-0918) showed nearly identical kinetics of K^+^ release to phage with the fully P22 needle protein (figure 5A). Since there is only one conservative amino acid sequence difference between the HS1 and Sf6 knobs (Ala305 in HS1 is Ser270 in Sf6; figure 2D), the significant difference in K^+^ release observed between UC-0918 and UC-0926 is likely due to the presence of the Sf6 shaft residues 69–141 in UC-0918 (which have a number of differences from P22 shaft residues); the mechanism underlying this difference could be due either some shaft function (such as interaction with the bacterial surface), or due to different allosteric coupling between possible conformational changes in the tip with changes that might be propagated through the needle to its N-terminus to effect needle release. We also performed K^+^ release measurements with P22 phages whose hybrid Sf6 needle knob domains carry the following alterations: Glu146Asp, Glu146Ala, Lys200Ala, Ser248Thr, Ser248Ala or Asp250Ala, as well as double mutants Glu146Ala/Asp250Ala, Lys200Ala/Asp250Ala and Glu146Ala/Lys200Ala. None of these mutants showed altered K^+^ release kinetics relative to the parental phage UC-0926 (figure 4E; data not shown for single mutants and the Glu146Ala/Asp250Ala phage UC-0931). In addition, K^+^ release was measured during infection by P22 UC-0926 containing the unaltered HS1 hybrid knob with and without 10 mM L-glutamate present in the cellular resuspension medium (KR buffer), and no significant difference in K^+^ release kinetics was observed (figure 4F) (virions were dialyzed for 24 hr at 4°C against several changes of TM buffer with no L-glutamate before use in this experiment). These results make it unlikely that externally added L-glutamate plays an important role in needle function during DNA delivery by these hybrid phages.

P22 virions in which the foldon replaces the C-terminal needle tip domain (UC-0927, above) showed much slower K^+^ release kinetics than P22 phages with wild type needles (figure 5A). This is paralleled by this phage’s tiny plaques and low PFU/particle ratio (above). Figure 5B shows that even at MOI values as high as 100, K^+^ release caused by phage P22 UC-0927 has a longer lag and lower 30 min extent of release than P22 with a wild type needle at an MOI of 10, so its slow K^+^ release cannot be explained simply by the low PFU/particle ratio (note that all the K^+^ release experiments presented here compare infections in which the MOIs are normalized to physical virion particles rather than PFUs; see table 2 and Materials and Methods).

While K^+^ release generally parallels the approximate period of phage DNA entry during the injection process, and in the case of T5 K^+^ release has two steps that appear to mirror its two stage DNA injection [42], [43], the mechanism of phage mediated K^+^ release is not known. It could be the result of opening of the channel for DNA transfer through the cytoplasmic membrane, and leakage through this channel, leakage around the outside of such a channel, or even through some other phage or host protein present in the membrane early in infection could allow K^+^ escape. Thus, although for ease of discussion above, we equated DNA entry and K^+^ release, we recognize that at present K^+^ release and DNA entry are only correlated observations, and they may not be mechanistically coupled.

### Concluding Remarks

In this work, we provide evidence that the distal tip of the tail needle is not required to confer host specificity on phage P22 and that the presence of bound L-glutamate in the Sf6 and HS1 hybrid needles does not affect P22 infection of *Salmonella* in the laboratory. It remains likely that in P22 and Sf6 the C-terminal needle domains do have evolutionarily important functions that are not easily measurable under laboratory conditions. For example, moderate differences in the speed of DNA delivery from the virion are not likely to limit the ability to form plaques. Thus, even if DNA release from the virion were slowed considerably (see below), it is possible that a significant overall slowing of the infectious cycle would not be evident. Nonetheless, the existence of functional virions that lack the native C-terminal tip of the needle has implications. It suggests that a DNA delivery model in which the needle tip domain makes an *essential* specific contact with a secondary receptor on the cell surface and then signals through the needle shaft to cause the needle’s N-terminal portal channel plug domain to release from the virion, thus opening the channel for DNA exit, is not completely correct.

Experiments with phages that have foldon-tipped and HS1 hybrid needles show that modifications of the tip of the phage P22 tail needle can affect the kinetics of DNA release during injection as measured by K^+^ release. Several non-mutually exclusive hypotheses for needle function could explain these results. For example, the needle tip could be part of the trigger that signals the virion to release its DNA, and modifying it could alters the kinetic of this signaling process. Although this may be true, the functionality of virions with the foldon tip indicates that the normal needle tip domain cannot be the only such trigger. Another, not mutually exclusive possibility is that the tail needle knob serves a mechanical function during genome ejection, comparable to the tip of a drill bit or hole punch in creating a hole in the inner membrane (the needle is released from the virion during DNA delivery [24], so it need not reach the inner membrane while still part of the virion). A larger globular domain at gp26 distal tip would then result in a “hole” of comparable diameter to the needle tip (∼45 Å in Sf6/HS1) (figure 3), and our crystallographic studies have shown that P22’s somewhat narrower ∼35 Å C-terminal tip can swing by ±18 degrees with respect to the helical shaft [27], [57], thus perhaps opening a hole larger than its diameter. Multiple copies of each of the P22 ejection proteins, gp7, gp16 and gp20, are also released from the virion during DNA delivery [24], and they are required for successful DNA entry into the cell cytoplasm. They are thought to perhaps build a structure or conduit that allows DNA transit from the virion through the membranes and periplasmic space into the cell [4]. Thus, if the needle protein in the membrane were to be replaced by the ejection proteins or were to create a hole that could then be occupied by the ejection proteins, the thinner foldon-tipped needle (diameter ∼25 Å; figure 3) might be more difficult for the ejection proteins to utilize.

In conclusion, whatever mechanisms P22-like phages have developed to efficiently eject their genomes into Gram negative bacteria, our data make a function of the tail needle distal tip in host specificity unlikely (although the possibility remains of a specific knob function during infection of *Shigella* by Sf6), and provide evidence in support a role of this tip in facilitating the kinetics of genome delivery during infection.

## Materials and Methods

### Bacterial Strains

*S. enterica* serovar Typhimurium LT2 strain UB-1790 (*leuA* ^–^414, r^–^, m^+^, Fels2^–^, *sup°* (P22 *sieA^–^*Δ1, *15^–^*ΔSC302::Kan^R^, *13^–^*amH101)) was used as the parent for alterations in the tail needle gene. The P22 prophage in this strain carries a kanamycin resistance gene to ensure prophage presence [58], a nonsense mutation in gene *13* to allow control of lysis by chloroform after induction to lytic growth [12], and the *sieA^–^*Δ1 deletion removes sequences that inhibit tailspike gene expression after induction but does not remove any essential genes [33], [59], [60]. The Kan^R^ insertion inactivates gene *15*, so titers were determined on LB plates containing 10 mM citrate [61]. The genotypes of the bacteria used in this study are given in table 2, and their construction is described below. Lytic growth of phages from lysogens was induced by addition of 0.5 µg/ml mitomycin C (Sigma, St. Louis, MO) and continued shaking at 37°C for 3–16 hr, and phage particles were titered on *S. enterica supE amber* mutant suppressing strain UB-0002 [62].

### Phage Strains

Two recombineering strategies were used in these manipulations, as described (i) by Karlinsey [63] in which the tetracycline resistance cassette TetRA is selected for by requiring growth in the presence of tetracycline, and loss of TetRA is selected for by growth in the presence of anhydrotetracyline hydrochloride (Thermo Fisher Scientific, Waltham, MA) [33], [58], and (ii) by Warming *et al.* [64] in which *galK^+^* insertions were selected by requiring growth on galactose as the sole carbon source and *galK^–^* bacteria were selected by growth in the presence of 2-deoxygalactose [33]. The phage lambda recombination function expressing plasmid pKD47 [65] was present during recombineering manipulations and was removed by growth at 42°C when it was no longer required. Electroporation was performed with a BIORAD Gene Pulser (25 uF, 2.4 KV, 200 W in 0.2 cm cuvettes). All genetic alterations were sequenced to confirm their structure.

P22 prophages that carry the C-terminal tail needle knob domain of phage Sf6 were constructed as follows: First a “recipient” prophage in which the TetRA cassette resides in P22 gene *26* was constructed. The TetRA cassette was amplified from DNA of strain UB-1737 (the kind gift of K. Hughes) using oligonucleotides A and B (table S1) and inserted by homologous recombination between codon 64 and the stop codon of the UB-1832 prophage gene *26* resulting in strain UB-1940. Sf6 needle gene DNA was PCR amplified from phage Sf6 clear mutant [66] DNA with oligonucleotides C and D that amplify Sf6 bp 8455–9102 (Accession No. AF547987) and have 3′ P22 sequence tails that allow the amplified fragment to replace P22 bp 84113–8909 when recombined into the prophage genome of UB-1940. The resulting strain UB-1918 carries a prophage in whose hybrid needle gene (*26::Sf6-3*) P22 codons 69–233 are replaced by phage Sf6 gene *9* codons 69 through 282 (Sf6 needle gene *9* is orthologous to P22 gene *26*). This replacement includes part of the coiled-coil shaft domain and all of the C-terminal knob domain. The HS1 knob replacement (hybrid gene *26::HS1-1*) was made in an analogous manner as follows: An *E. coli galK* gene expression cassette was inserted into gene *26* of the prophage of strain UB-1790 by recombineering using DNA amplified from plasmid pGalK [64] (the kind gift of Don Court) with primers E and F whose P22 sequence 5′-tails result in the recombinational replacement of P22 gene *26* codons 142–233 by the *galK* gene and selection for growth on galactose. The resulting strain is UB-2078. DNA amplified from *E. coli* HS (UB-1732) DNA with primers G and H was used to replace the *galK* gene of UB-2078 so that codons 174 to 317 of the phage HS1 needle gene (locus_tag EcHS_A0316) replace P22 needle codons 141–233 to give strain UB-2083.

Modifications of the UB-1918 prophage (above) that contain point mutations in the L-glutamate binding site of the Sf6 needle knob were constructed as follows: A TetRA cassette was inserted into the P22 prophage of strain UB-1918 in three different places so that it replaces (i) codons 145 and 146 (primers I and J) in the 282 codon long, hybrid needle gene to create strain UB-1942, (ii) codon 200 (primers K and L and strain UB-1943) or (iii) codons 248 and 249 (primers M and N and strain UB-1944). Plasmid pPP304 carries the whole Sf6 *9* gene cloned into plasmid pET15b (Novagen, EMD Biosciences, Darmstadt, Germany), and single codon mutations were created in gene *9* of this plasmid with the QUICKCHANGE® site directed mutagenesis kit, Pfu Ultra Polymerase (Stratagene, La Jolla, CA), and restriction enzyme *Dpn*I as recommended by the manufacturer (New England Biolabs, Ipswich, MA). These modified plasmid genes were amplified with primers O and P and the resulting DNA was used to replace the TetRA cassette in UB-1942, -1943 or -1944. The changes made in these strains (UB-1919 through UB-1929) are indicated in table 2.

A prophage in which C-terminal codons 141–233 of P22 gene *26* are replaced by DNA encoding the T4 fibritin foldon [38], [39] was constructed as follows: An *E. coli galK* gene expression cassette was amplified from plasmid pGalK [64] using primers Q and R whose P22 sequence 5′-tails result in the recombinational replacement of P22 bp 8403–8913 (gene *26* codons 65 through the stop codon) after electroporation into UB-1790 and selection to be *galK^+^*. The *galK* gene of the resulting strain (UB-1807) was then replaced by DNA amplified from plasmid pMAL-PP-gp26(1–140)-F [39] with primers S and T; this plasmid carries a P22 gene *26* in which codons 141–233 are replaced by the 25 foldon codons, and the resulting prophage of strain UB-1941 carries this modified gene *26*.

### Potassium Ion Efflux Measurement

Potassium ion concentrations were measured using an Orion Ionplus potassium electrode (Thermo Scientific) and a Corning model 430 pH meter. *Salmonella* cells were grown to 2×10^8^ cells/ml in LB broth [67], spun down, washed once in KR buffer (10 mM NaPO_4_ buffer, pH = 7.4, 100 mM NaCl, 10 mM MgSO_4_) and finally resuspended at their initial concentration in KR buffer; no difference was observed between the two host strains UB-0001 or UB-0002. Aliquots of concentrated stocks of phage particles (>10^13^/ml) that had been purified by CsCl step gradient centrifugation [68] and dialyzed against TM (10 mM TrisCl, pH 7.5, 1 mM MgCl_2_) were used to infect the cells. Ten ml of the cell suspension was equilibrated to the desired temperature, the electrode was inserted into the cell suspension, phages were added 5 min later from concentrated stocks, and the mixture was briefly vortexed. The concentration of released potassium ions was monitored for 20–60 min after infection, and after these measurements the cells were lysed with Bugbuster reagent (EMD Millipore) or by boiling for 10 min, and total released K^+^ was measured. The infections in each panel of figures 4 and 5 were performed on the same batch of cells. Some of the mutant phages used have somewhat different PFU/particle ratios (table 3), and it is unclear whether comparisons between phages should be done with equal numbers of PFU’s or physical particles. The reason for these PFU/particle differences are not known, but since the virion proteins appear to be present in the same numbers in the different phages (in particular tailspike appears to be the same) there is no reason to believe that some particles are physically different from others in any given phage genotype. We performed comparative experiments both ways (equal numbers of PFUs or equal numbers of particles) and obtained results that gave the same qualitative conclusions. The data was more reproducible with equal numbers of physical particles, so all the K^+^ release curves presented here were obtained this manner, and MOIs were calculated assuming a PFU/particle ratio of one for “wild type” P22 (UC-0911) and the PFU/particle ratios in table 3.

### Cloning, Expression and Purification of HS1 Knob

The gene coding prophage HS1 tail needle knob (residues 167–317) was PCR amplified from strain UB-1732 DNA (locus_tag EcHS_A0316, Accession No. CP00802) and cloned in a pMal-c2e expression vector (New England Biolabs) between restriction sites XbaI and HindIII (plasmid pMal-HS1-knob). This plasmid was expressed in *E. coli* BL21(DE3) pLysE at 37°C as follows: Cells were grown to A_595_ = 0.6, the culture was induced for 16 h at 22°C by the addition of 0.5 mM isopropyl 1-thio-β-D-galactopyranoside, and the resulting cells were lysed by sonication in lysis buffer (20 mM Tris-HCl pH 8.0, 250 mM NaCl). The protein containing the HS1 knob fused to an N-terminal maltose binding protein (MBP-HS1) was purified by Amylose affinity chromatography (New England Biolabs). The fusion protein was digested with PreScission protease (GE Healthcare) and the resulting free HS1 knob protein was purified by Superdex 200 size exclusion chromatography (GE Healthcare) followed by passage over a 5 ml DEAE column (Sigma) that captured the MBP; pure HS1 tail needle knob was recovered in the DEAE column flow-through. The purified HS1 knob protein (∼51 kDa) was concentrated to 10 mg/ml in 20 mM Tris-HCl pH 8.0, 50 mM NaCl using Sartorius ultracentrifugal filter device with a 10,000 Da molecular weight cutoff.

### Crystallization and Structure Determination of HS1 Knob

HS1 knob was crystallized by mixing equal volumes of the protein solution and of a reservoir solution composed of 26% PEG 3350, 50 mM Tris-HCl pH 8.0 using the hanging drop vapor diffusion method. Crystals grew to full size over 2 weeks, were cryoprotected by quickly soaking in reservoir solutions also containing 27% ethylene glycol, and were flash-frozen in liquid nitrogen. Diffraction data were collected at CHESS F1 and NSLS X6A beamlines. HS1 knob data were processed with the HKL2000 software package [69]. These crystals belong to space group P212121 and diffracted to 1.1 Å resolution (table 1). The structure was solved by molecular replacement with Phaser using the Sf6 tail needle knob (pdb code 3RWN) as search model [70]. One copy of trimeric HS1 knob was present in the asymmetric unit. The structure was built with COOT software [71] and refinement procedures were carried out using Phenix.refine [72]. The structure was validated by MolProbity [73] and analyzed by the EBI-PISA server [74] for interface interactions. All 151 residues of HS1 knob were unambiguously traced in the electron density map. The final model also includes 713 water molecules, one phosphate ion at distal tip of knob and three L-glutamate molecules at the dimeric interfaces formed by knob homotrimer (table 1). The coordinates and structure factors for HS1 tail needle knob have been deposited in the protein Data Bank with accession code 4K6B.

## Supporting Information

Figure S1 Relationship of host bacterial species to P22-like phage tail needle tip domain type.
(PDF)

Figure S2 Relationships among the phage Sf6 type tail needle C-terminal domains of the P22-like phages.
(PDF)

Figure S3 Conserved residues in Sf6 tail needle knob-like proteins.
(PDF)

Figure S4 The hybrid P22:HS1-1 hybrid needle is incorporated into the virion.
(PDF)

Table S1 Oligonucleotides used in this study.
(PDF)
