# Supplementary material for: The Tip of the Tail Needle Affects the Rate of DNA Delivery by Bacteriophage P22
Source: PLoS One. 2013 Aug 12;8(8):e70936. doi: 10.1371/journal.pone.0070936 (PMC3741392; doi:10.1371/journal.pone.0070936)
Supplement: Figure S1 — Relationship of host bacterial species to P22-like phage tail needle tip domain type. (PDF) [file pone.0070936.s001.pdf]

Figure S1

Leavitt *et al.*

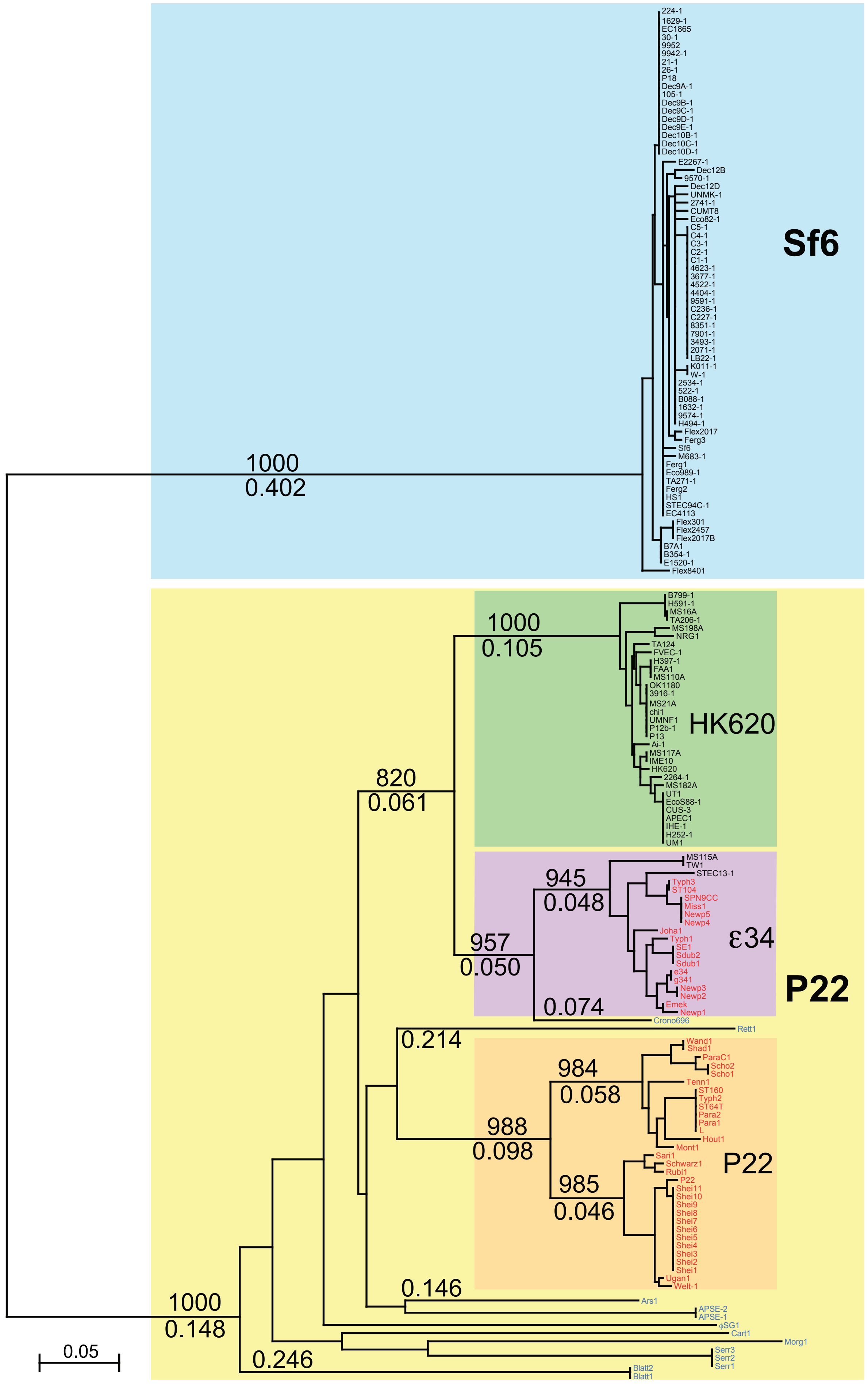

Figure S1. Relationship of host bacterial species to P22-like phage tail needle tip domain type.

A neighbor joining tree (created with Clustal X2 [Larkin MA, Blackshields G, Brown NP, Chenna R, McGettigan PA, *et al.* (2007) Clustal W and Clustal X version 2.0. Bioinformatics 23: 2947-2948]) is shown with selected branch lengths (numbers between 0. and 1) and bootstrap values out of 1000 trials (between 1 and 1000). Bootstrap values for the nodes that are not well-supported are not shown. A scale in fractional difference is shown in the lower left. The Sf6 type domains are highlighted with a large blue box and P22 type domains with a large yellow box; these two domain types are not homologous but are combined in this tree to demonstrate the large sequence distance between them. *E. coli, Escherichia fergusonii* and *Shigella* phages and prophages are named in red text and *Salmonella enterica* phages and prophages are shown in black text. Other host species are shown in blue text. The names of these other hosts are given in the legend of figure 1, except for Crono696-1, which is a prophage in the genome of *Cronobacter sakazakii* 696. Prophage details can be obtained from the corresponding author.
