# Supplementary material for: The Tip of the Tail Needle Affects the Rate of DNA Delivery by Bacteriophage P22
Source: PLoS One. 2013 Aug 12;8(8):e70936. doi: 10.1371/journal.pone.0070936 (PMC3741392; doi:10.1371/journal.pone.0070936)
Supplement: Figure S2 — Relationships among the phage Sf6 type tail needle C-terminal domains of the P22-like phages. (PDF) [file pone.0070936.s002.pdf]

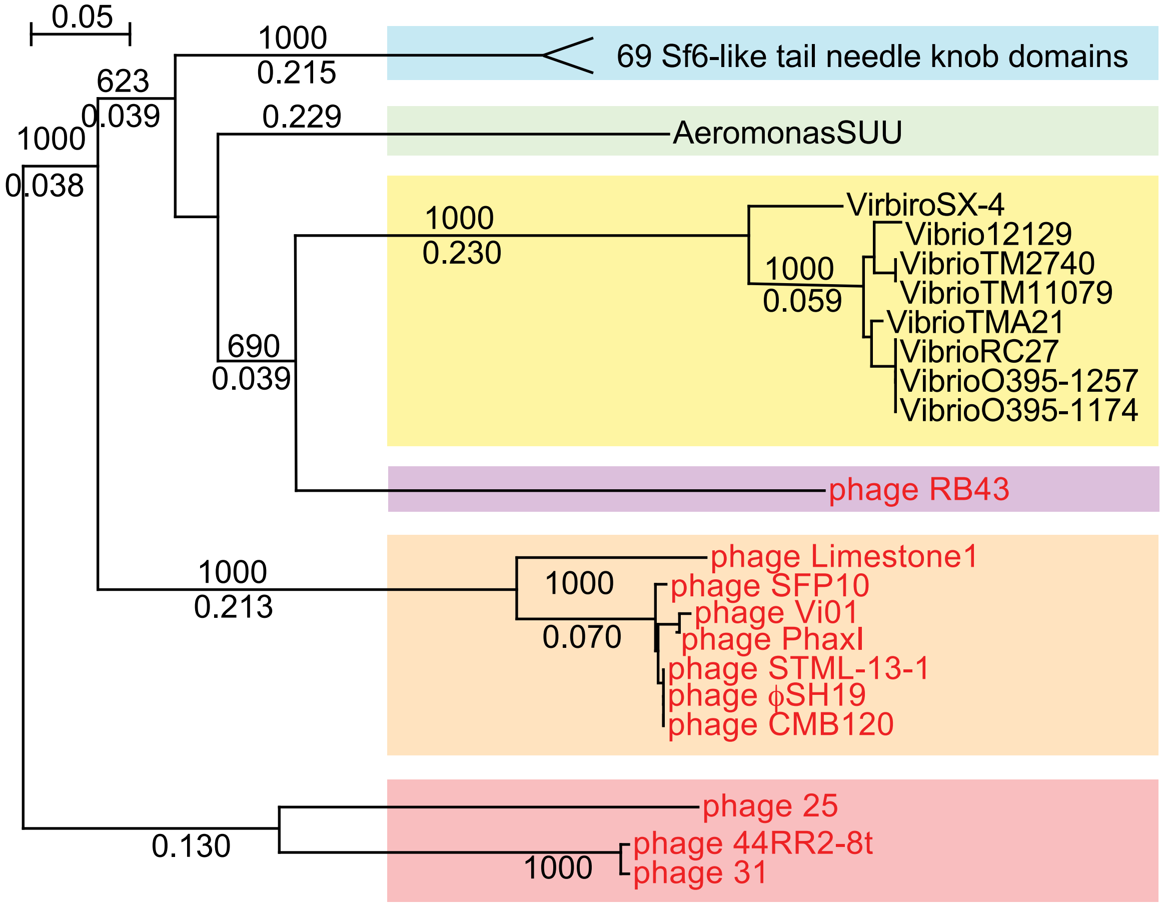

Figure S2. Relationships among the phage Sf6 type tail needle C-terminal domains of the P22-like phages.

A neighbor joining tree (created with Clustal X2 [Larkin et al. (2007) Clustal W and Clustal X version 2.0. Bioinformatics 23: 2947-2948]) is shown with selected branch lengths (numbers between 0. and 1) and bootstrap values out of 1000 trials (between 1 and 1000). Bootstrap values for the nodes that are not well-supported are not shown. A scale in fractional difference is shown in the upper left. The six major sequence types are highlighted with colored boxes. Bone fide bacteriophages are labeled in red and prophages in black. Prophage names are provisional and some prophage names were reported in Casjens and Thuman-Commike [(2011) Evolution of mosaically related tailed bacteriophage genomes seen through the lens of phage P22 virion assembly. Virology 411: 393-415]. Prophage details can be obtained from the corresponding author.
