# Supplementary material for: The Tip of the Tail Needle Affects the Rate of DNA Delivery by Bacteriophage P22
Source: PLoS One. 2013 Aug 12;8(8):e70936. doi: 10.1371/journal.pone.0070936 (PMC3741392; doi:10.1371/journal.pone.0070936)
Supplement: Figure S3 — Conserved residues in Sf6 tail needle knob-like proteins. (PDF) [file pone.0070936.s003.pdf]

```
Flex2017B          ------------------------------------------------------------
Flex2457           ------------------------------------------------------------
Flex301            ------------------------------------------------------------
FlexK-304          ------------------------------------------------------------
FlexK-671          ------------------------------------------------------------
Flex2930-71        ------------------------------------------------------------
Flex2747-71        ------------------------------------------------------------
E1520-1            ------------------------------------------------------------
B354-1             ------------------------------------------------------------
B7A1               ------------------------------------------------------------
Boyd-1             ------------------------------------------------------------
Ferg3              ------------------------------------------------------------
Flex2017           ------------------------------------------------------------
9570-1             ------------------------------------------------------------
Dec12B             ------------------------------------------------------------
9574-1             ------------------------------------------------------------
W-1                ------------------------------------------------------------
K011-1             ------------------------------------------------------------
H494-1             ------------------------------------------------------------
1632-1             ------------------------------------------------------------
B088-1             ------------------------------------------------------------
522-1              ------------------------------------------------------------
2534-1             ------------------------------------------------------------
FlexK-404          ------------------------------------------------------------
Eco82-1            ------------------------------------------------------------
UMNK-1             ------------------------------------------------------------
CUMT8              ------------------------------------------------------------
2741-1             ------------------------------------------------------------
LB22-1             ------------------------------------------------------------
2071-1             ------------------------------------------------------------
3493-1             ------------------------------------------------------------
7901-1             ------------------------------------------------------------
8351-1             ------------------------------------------------------------
C227-1             ------------------------------------------------------------
C236-1             ------------------------------------------------------------
9591-1             ------------------------------------------------------------
4404-1             ------------------------------------------------------------
4522-1             ------------------------------------------------------------
3677-1             ------------------------------------------------------------
4623-1             ------------------------------------------------------------
C1-1               ------------------------------------------------------------
C2-1               ------------------------------------------------------------
C3-1               ------------------------------------------------------------
C4-1               ------------------------------------------------------------
C5-1               ------------------------------------------------------------
Dec12D             ------------------------------------------------------------
Sf6                ------------------------------------------------------------*
EC4113             ------------------------------------------------------------
STEC94C-1          ------------------------------------------------------------
Ferg2              ------------------------------------------------------------
TA271-1            ------------------------------------------------------------
Eco989-1           ------------------------------------------------------------
Ferg1              ------------------------------------------------------------
HS1                ------------------------------------------------------------*
M863-1             ------------------------------------------------------------
E1167-1            ------------------------------------------------------------
Dec10C-1           ------------------------------------------------------------
Dec10D-1           ------------------------------------------------------------
Dec10B-1           ------------------------------------------------------------
Dec9E-1            ------------------------------------------------------------
Dec9D-1            ------------------------------------------------------------
Dec9C-1            ------------------------------------------------------------
Dec9B-1            ------------------------------------------------------------
105-1              ------------------------------------------------------------
Dec9A-1            ------------------------------------------------------------
P18                ------------------------------------------------------------
30-1               ------------------------------------------------------------
9952               ------------------------------------------------------------
EC1865             ------------------------------------------------------------
1629-1             ------------------------------------------------------------
224-1              ------------------------------------------------------------
26-1               ------------------------------------------------------------
21-1               ------------------------------------------------------------
9942-1             ------------------------------------------------------------
Flex8401           ------------------------------------------------------------
FlexM90T           ------------------------------------------------------------
phage31            MILNPKAVLNNTSSGSSGVSSFNGRIGPVDPESGDYTADMVNAIEKAPTDSQRRVLIGTT 60
44RR2-8torf183     MILNPKAVLNNTSSGSSGVSSFNGRIGPVDPESGDYTADMVNAIEKAPTDSQRRVLIGTT 60
CMB120orf226       ------------------------------------------------------------
phiHS19            ------------------------------------------------------------
STML-13-1          ------------------------------------------------------------
PhaxI              ------------------------------------------------------------
Vi01               ------------------------------------------------------------
SFP10              ------------------------------------------------------------
Limestone1         ------------------------------------------------------------
AeromonasSUU       ------------------------------------------------------------
RB43               -------------------------------------------------------MLISA 5
Vibrio0395-1174    ------------------------------------------------------------
Vibrio0395-1257    ------------------------------------------------------------
VibrioRC27         ------------------------------------------------------------
VibrioTMA21        ------------------------------------------------------------
VibrioTM11079      ------------------------------------------------------------
VibrioTM2740       ------------------------------------------------------------
Vibrio12129        ------------------------------------------------------------
VirbiroSX-4        ------------------------------------------------------------
```

```
Flex2017B           ------------------------------------------------------------
Flex2457            ------------------------------------------------------------
Flex301             ------------------------------------------------------------
FlexK-304           ------------------------------------------------------------
FlexK-671           ------------------------------------------------------------
Flex2930-71         ------------------------------------------------------------
Flex2747-71         ------------------------------------------------------------
E1520-1             ------------------------------------------------------------
B354-1              ------------------------------------------------------------
B7A1                ------------------------------------------------------------
Boyd-1              ------------------------------------------------------------
Ferg3               ------------------------------------------------------------
Flex2017            ------------------------------------------------------------
9570-1              ------------------------------------------------------------
Dec12B              ------------------------------------------------------------
9574-1              ------------------------------------------------------------
W-1                 ------------------------------------------------------------
K011-1              ------------------------------------------------------------
H494-1              ------------------------------------------------------------
1632-1              ------------------------------------------------------------
B088-1              ------------------------------------------------------------
522-1               ------------------------------------------------------------
2534-1              ------------------------------------------------------------
FlexK-404           ------------------------------------------------------------
Eco82-1             ------------------------------------------------------------
UMNK-1              ------------------------------------------------------------
CUMT8               ------------------------------------------------------------
2741-1              ------------------------------------------------------------
LB22-1              ------------------------------------------------------------
2071-1              ------------------------------------------------------------
3493-1              ------------------------------------------------------------
7901-1              ------------------------------------------------------------
8351-1              ------------------------------------------------------------
C227-1              ------------------------------------------------------------
C236-1              ------------------------------------------------------------
9591-1              ------------------------------------------------------------
4404-1              ------------------------------------------------------------
4522-1              ------------------------------------------------------------
3677-1              ------------------------------------------------------------
4623-1              ------------------------------------------------------------
C1-1                ------------------------------------------------------------
C2-1                ------------------------------------------------------------
C3-1                ------------------------------------------------------------
C4-1                ------------------------------------------------------------
C5-1                ------------------------------------------------------------
Dec12D              ------------------------------------------------------------
Sf6                 ------------------------------------------------------------
EC4113              ------------------------------------------------------------*
STEC94C-1           ------------------------------------------------------------
Ferg2               ------------------------------------------------------------
TA271-1             ------------------------------------------------------------
Eco989-1            ------------------------------------------------------------
Ferg1               ------------------------------------------------------------
HS1                 ------------------------------------------------------------*
M863-1              ------------------------------------------------------------
E1167-1             ------------------------------------------------------------
Dec10C-1            ------------------------------------------------------------
Dec10D-1            ------------------------------------------------------------
Dec10B-1            ------------------------------------------------------------
Dec9E-1             ------------------------------------------------------------
Dec9D-1             ------------------------------------------------------------
Dec9C-1             ------------------------------------------------------------
Dec9B-1             ------------------------------------------------------------
105-1               ------------------------------------------------------------
Dec9A-1             ------------------------------------------------------------
P18                 ------------------------------------------------------------
30-1                ------------------------------------------------------------
9952                ------------------------------------------------------------
EC1865              ------------------------------------------------------------
1629-1              ------------------------------------------------------------
224-1               ------------------------------------------------------------
26-1                ------------------------------------------------------------
21-1                ------------------------------------------------------------
9942-1              ------------------------------------------------------------
Flex8401            ------------------------------------------------------------
FlexM90T            ------------------------------------------------------------
phage31             PTVEELIDPVTVVDNLTSDSSNSALSAKQGKLLQDGKQPTITGAASTIASVDLTPVRIAA 120
44RR2-8torf183      PTVEELIDPITVVDNLTSDSSTSALSAKQGKLLQDGKQPTITGAASTIASVDLTPVRIAA 120
CMB120orf226        ------------------------------------------------------------
phiHS19             -------------------------------------------------------MAILT 5
STML-13-1           ------------------------------------------------------------
PhaxI               -------------------------------------------------------MAILT 5
Vi01                -------------------------------------------------------MAILT 5
SFP10               -------------------------------------------------------MAILT 5
Limestone1          -------------------------------------------------------MAILP 5
AeromonasSUU        ---------------------------------------------------------MPF 3
RB43                SSVDELNSKVNEMLSQGMYLWGSPFVVPSDYLNQTRFFQQVSNVNPSSGGTDGKSAYQLW 65
VibrioO395-1174     ---------------------------------------------------------MIY 3
VibrioO395-1257     ---------------------------------------------------------MIY 3
VibrioRC27          ---------------------------------------------------------MIY 3
VibrioTMA21         ---------------------------------------------------------MIY 3
VibrioTM11079       ---------------------------------------------------------MIY 3
VibrioTM2740        ---------------------------------------------------------MIY 3
Vibrio12129         ---------------------------------------------------------MIY 3
VirbiroSX-4         ---------------------------------------------------------MIY 3
```

```
Flex2017B            -----------------------TTRKKSEVVYS-GVSVTIP--TAPTNLVSLLKTL-TP 33
Flex2457             -----------------------TTRKKSEVVYS-GVSVTIP--TAPTNLVSLLKTL-TP 33
Flex301              -----------------------TTRKKSEVVYS-GVSVTIP--TAPTNLVSLLKTL-TP 33
FlexK-304            -----------------------TTRKKSEVVYS-GVSVTIP--TAPTNLVSLLKTL-TP 33
FlexK-671            -----------------------TTRKKSEVVYS-GVSVTIP--TAPTNLVSLLKTL-TP 33
Flex2930-71          -----------------------TTRKKSEVVYS-GVSVTIP--TAPTNLVSLLKTL-TP 33
Flex2747-71          -----------------------TTRKKSEVVYS-GVSVTIP--TAPTNLVSLLKTL-TP 33
E1520-1              -----------------------TTRKKSEVVYS-GVSVTIP--TAPTNLVSLLKTL-TP 33
B354-1               -----------------------TTRKKSEVVYS-GVSVTIP--TAPTNLVSLLKTL-TP 33
B7A1                 -----------------------TTRKKSEVVYS-GVSVTIP--TAPTNLVSLLKTL-TP 33
Boyd-1               -----------------------TTRKKSEVVYS-GVSVTIP--TAPTNLVSLLKTL-TP 33
Ferg3                -----------------------TTRKKSEVVYS-GVSVTIP--TAPTNLVSLLKTL-TP 33
Flex2017             -----------------------TTRKKSEVVYS-GVSVTIP--TAPTNLVSLLKTL-TP 33
9570-1               -----------------------TARKKSEVVYS-GVSVTIP--IAPTNLVSLLKTL-TP 33
Dec12B               -----------------------TARKKSEVVYS-GVSVTIP--IAPTNLVSLLKTL-TP 33
9574-1               -----------------------TARKKSEVVYS-GVSVTIP--TAPTNLVSLLKTL-TP 33
W-1                  -----------------------TTRKKSEVVYS-GVSVTIP--TAPTNLVILLKTL-TP 33
K011-1               -----------------------TTRKKSEVVYS-GVSVTIP--TAPTNLVILLKTL-TP 33
H494-1               -----------------------TTRKKSEVVYS-GVSVTIP--TAPTNLVSLLKTL-TP 33
1632-1               -----------------------TTRKKSEVVYS-GVSVTIP--TAPTNLVSLLKTL-TP 33
B088-1               -----------------------TTRKKSEVVYS-GVSVTIP--TAPTNLVSLLKTL-TP 33
522-1                -----------------------TTRKKSEVVYS-GVSVTIP--TAPTNLVSLLKTL-TP 33
2534-1               -----------------------TTRKKSEVVYS-GVSVTIP--TAPTNLVSLLKTL-TP 33
FlexK-404            -----------------------TTRKKSEVVYS-GVSVTIP--TAPTNLVSLLKTL-TP 33
Eco82-1              -----------------------TTRKKSEVVYS-GVSVTIP--TAPTNLVSLLKTL-TP 33
UMNK-1               -----------------------TTRKKSEVVYS-GVSVTIP--TAPTNLVSLLKTL-TP 33
CUMT8                -----------------------TTRKKSEVVYS-GVSVTIP--TAPTNLVSLLKTF-TP 33
2741-1               -----------------------TTRKKSEVVYS-GVSVTIP--TAPTNLVSLLKTL-TP 33
LB22-1               -----------------------TTRKKSEVVYS-GVSVTIP--TAPTNLVSLLKTL-TP 33
2071-1               -----------------------TTRKKSEVVYS-GVSVTIP--TAPTNLVSLLKTL-TP 33
3493-1               -----------------------TTRKKSEVVYS-GVSVTIP--TAPTNLVSLLKTL-TP 33
7901-1               -----------------------TTRKKSEVVYS-GVSVTIP--TAPTNLVSLLKTL-TP 33
8351-1               -----------------------TTRKKSEVVYS-GVSVTIP--TAPTNLVSLLKTL-TP 33
C227-1               -----------------------TTRKKSEVVYS-GVSVTIP--TAPTNLVSLLKTL-TP 33
C236-1               -----------------------TTRKKSEVVYS-GVSVTIP--TAPTNLVSLLKTL-TP 33
9591-1               -----------------------TTRKKSEVVYS-GVSVTIP--TAPTNLVSLLKTL-TP 33
4404-1               -----------------------TTRKKSEVVYS-GVSVTIP--TAPTNLVSLLKTL-TP 33
4522-1               -----------------------TTRKKSEVVYS-GVSVTIP--TAPTNLVSLLKTL-TP 33
3677-1               -----------------------TTRKKSEVVYS-GVSVTIP--TAPTNLVSLLKTL-TP 33
4623-1               -----------------------TTRKKSEVVYS-GVSVTIP--TAPTNLVSLLKTL-TP 33
C1-1                 -----------------------TTRKKSEVVYS-GVSVTIP--TAPTNLVSLLKTL-TP 33
C2-1                 -----------------------TTRKKSEVVYS-GVSVTIP--TAPTNLVSLLKTL-TP 33
C3-1                 -----------------------TTRKKSEVVYS-GVSVTIP--TAPTNLVSLLKTL-TP 33
C4-1                 -----------------------TTRKKSEVVYS-GVSVTIP--TAPTNLVSLLKTL-TP 33
C5-1                 -----------------------TTRKKSEVVYS-GVSVTIP--TAPTNLVSLLKTL-TP 33
Dec12D               -----------------------TTRKKSEVVYS-GVSVTIP--TAPTNLVSLLKTL-TP 33
Sf6                  -----------------------TTRKKSEVVYS-GVSVTIP--TAPTNLVSLLKTL-TP 33*
EC4113               -----------------------TTRKKSEVVYS-GVSVTIP--TAPTNLVSLLKTL-TP 33
STEC94C-1            -----------------------TTRKKSEVVYS-GVSVTIP--TAPTNLVSLLKTL-TP 33
Ferg2                -----------------------TTRKKSEVVYS-GVSVTIP--TAPTNLVSLLKTL-TP 33
TA271-1              -----------------------TTRKKSEVVYS-GVSVTIP--TAPTNLVSLLKTL-TP 33
Eco989-1             -----------------------TTRKKSEVVYS-GVSVTIP--TAPTNLVSLLKTL-TP 33
Ferg1                -----------------------TTRKKSEVVYS-GVSVTIP--TAPTNLVSLLKTL-TP 33
HS1                  -----------------------TTRKKSEVVYS-GVSVTIP--TAPTNLVSLLKTL-TP 33*
M863-1               -----------------------TTRKKSEVVYS-GVSVTIP--TAPTNLVSLLKTL-TP 33
E1167-1              -----------------------TTRKKSEVVYS-GVSVTIP--TAPTNLVSLLKTL-TP 33
Dec10C-1             -----------------------TTRKKSEVVYS-GVSVTIP--TAPTNLVSLLKTL-TP 33
Dec10D-1             -----------------------TTRKKSEVVYS-GVSVTIP--TAPTNLVSLLKTL-TP 33
Dec10B-1             -----------------------TTRKKSEVVYS-GVSVTIP--TAPTNLVSLLKTL-TP 33
Dec9E-1              -----------------------TTRKKSEVVYS-GVSVTIP--TAPTNLVSLLKTL-TP 33
Dec9D-1              -----------------------TTRKKSEVVYS-GVSVTIP--TAPTNLVSLLKTL-TP 33
Dec9C-1              -----------------------TTRKKSEVVYS-GVSVTIP--TAPTNLVSLLKTL-TP 33
Dec9B-1              -----------------------TTRKKSEVVYS-GVSVTIP--TAPTNLVSLLKTL-TP 33
105-1                -----------------------TTRKKSEVVYS-GVSVTIP--TAPTNLVSLLKTL-TP 33
Dec9A-1              -----------------------TTRKKSEVVYS-GVSVTIP--TAPTNLVSLLKTL-TP 33
P18                  -----------------------TTRKKSEVVYS-GVSVTIP--TAPTNLVSLLKTL-TP 33
30-1                 -----------------------TTRKKSEVVYS-GVSVTIP--TAPTNLVSLLKTL-TP 33
9952                 -----------------------TTRKKSEVVYS-GVSVTIP--TAPTNLVSLLKTL-TP 33
EC1865               -----------------------TTRKKSEVVYS-GVSVTIP--TAPTNLVSLLKTL-TP 33
1629-1               -----------------------TTRKKSEVVYS-GVSVTIP--TAPTNLVSLLKTL-TP 33
224-1                -----------------------TTRKKSEVVYS-GVSVTIP--TAPTNLVSLLKTL-TP 33
26-1                 -----------------------TTRKKSEVVYS-GVSVTIP--TAPTNLVSLLKTL-TP 33
21-1                 -----------------------TTRKKSEVVYS-GVSVTIP--TAPTNLVSLLKTL-TP 33
9942-1               -----------------------TTRKKSEVVYS-GVSVTIP--TAPTNLVSLLKTL-TP 33
Flex8401             -----------------------TTRKKSEVVYS-GVSVTIP--TAPTNLVSLLKTL-TP 33
FlexM90T             -----------------------TTRKKSEVVYS-GVSVTIP--TAPTNLVSLLKTL-TP 33
phage31              TDSNGKMTTSLVSVSDLDLIKDRTTRVKSELEWT-GLNIDVT--GTALNLVGALKAI-TP 176
44RR2-8torf183       TDSNGKMTTSLVSVSDLDLIKDRTTRVKSELEWT-GLNIDVT--GTALNLVGALKAI-TP 176
CMB120orf226         ------MLQ--------------THRIKTEVRFS-GLSQLLTSGATGIDLLTVLDGK-TP 38
phiHS19              SPYLGNMLQ--------------THRIKTEVRFS-GLSQLLTSGATGIDLLTVLDGK-TP 49
STML-13-1            ------MLQ--------------THRIKTEVRFS-GLSQLLTSGATGIDLLTVLDGK-TP 38
PhaxI                SPYLGNMLQ--------------THRIKTEVRFS-GLSQLLTSGATGIDLLTVLDGK-TP 49
Vi01                 SPYLGNMLQ--------------THRIKTEVRFS-GLSQLLTSGATGIDLLTVLDGK-TP 49
SFP10                SPYLGNMLQ--------------THRIKTEVRFS-GLSQLLTSGATGIDLLTVLDGK-TP 49
Limestone1           SPYLGNMLQ--------------THRIKVEVRFS-GLTAALPAGATGVDLLTLLDGK-TP 49
AeromonasSUU         GKAFVYQAP---------------KRKKTEVFWT-GLTGVVLTADTDYNLVTLLKGLPAP 47
RB43                 VEQPGNEGKTLDDFFDS----IAGVRKKSEVFWT-GLSLVIP-EDTPTNFINLIKGT-IP 118
Vibrio0395-1174      EAPLTSGSD--------------AHRHKSEVLFDFSASPLVFTQGVTYNLIDRIKAA-AP 48
Vibrio0395-1257      EAPLTSGSD--------------AHRHKSEVLFDFSASPLVFTQGVTYNLIDRIKAA-AP 48
VibrioRC27           EAPLTSGSD--------------AHRHKSEVLFDFSASPLVFTQGVTYNLIDRIKAA-AP 48
VibrioTMA21          EAPLTSGSD--------------APRHKSEVLFDFSASPLVFTQGVTYNLIDRIKAA-AP 48
VibrioTM11079        EAPLTSGSD--------------APRHKSEVLFDFSASPLVFTQGVTYNLIDRIKAT-AP 48
VibrioTM2740         EAPLTSGSD--------------APRHKSEVLFDFSASPLVFTQGVTYNLIDRIKAT-AP 48
Vibrio12129          EAPLTSGSD--------------APRRKSEVLFDFSASPLVFTQGVTYNLIDRIKAT-VP 48
VirbiroSX-4          EAPLIPVSD--------------APRRKSEVLFDFSASPLVFTQGVTYNLIDRIKAR-TP 48
                                              * * *: :  . .  :    .  :::  :.    *
```

```
Flex2017B            SSGS---LAPFFDTVNNKMVVF-NENKTLLFKLSIVGTWP-SGTANRSMQLTFS-GSVPD 87
Flex2457             SSGS---LAPFFDTVNNKMVVF-NENKTLLFKLSIVGTWP-SGTANRSMQLTFS-GSVPD 87
Flex301              SSGS---LAPFFDTVNNKMVVF-NENKTLLFKLSIVGTWP-SGTANRSMQLTFS-GSVPD 87
FlexK-304            SSGS---LAPFFDTVNNKMVVF-NENKTLLFKLSIVGTWP-SGTANRSMQLTFS-GSVPD 87
FlexK-671            SSGS---LAPFFDTVNNKMVVF-NENKTLLFKLSIVGTWP-SGTANRSMQLTFS-GSVPD 87
Flex2930-71          SSGS---LAPFFDTVNNKMVVF-NENKTLLFKLSIVGTWP-SGTANRSMQLTFS-GSVPD 87
Flex2747-71          SSGS---LAPFFDTVNNKMVVF-NENKTLLFKLSIVGTWP-SGTANRSMQLTFS-GSVPD 87
E1520-1              SSGS---LAPFFDTVNNKMVVF-NENKTLFFKLSIVGTWP-SGTANRSMQLTFS-GSVPD 87
B354-1               SSGS---LAPFFDTVNNKMVVF-NENKTLFFKLSIVGTWP-SGTANRSMQLTFS-GSVPD 87
B7A1                 SSGS---LAPFFDTVNNKMVVF-NENKTLFFKLSIVGTWP-SGTANRSMQLTFS-GSVPD 87
Boyd-1               SSGS---LAPFFDTVNNKMVVF-NENKTLFFKLSIVGTWP-SGTANRSMQLTFS-GSVPD 87
Ferg3                SSGT---LAPFFDTVNNKMVVF-NENKTLFFKLSIVGTWP-SGTANRSMQLTFS-GSVPD 87
Flex2017             SSGT---LAPFFDTVNNKMVVF-NENKTLFFKLSIVGTWP-SGTANRSMQLTFS-GSVPD 87
9570-1               SSGT---LAPFFDTVNNKMVVF-NENKTLFFKLSIVGTWP-SGTANRSMQLTFS-GSVPD 87
Dec12B               SSGT---LAPFFDTVNNKMVVF-NENKTLFFKLSIVGTWP-SGTANRSMQLTFS-GSVPD 87
9574-1               SSGT---LAPFFDTVNNKMVVF-NENKTLFFKLSIVGTWP-SGTANRSMQLTFS-GSVPD 87
W-1                  SSGT---LAPFFDTVNNKMVVF-NENKTLFFKLSIVGTWP-SGTANRSMQLTFS-GSVPD 87
K011-1               SSGT---LAPFFDTVNNKMVVF-NENKTLFFKLSIVGTWP-SGTANRSMQLTFS-GSVPD 87
H494-1               SSGT---LAPFFDTVNNKMVVF-NENKTLFFKLSIVGTWP-SGTANRSMQLTFS-GSVPD 87
1632-1               SSGT---LAPFFDTVNNKMVVF-NENKTLFFKLSIVGTWP-SGTANRSMQLTFS-GSVPD 87
B088-1               SSGT---LAPFFDTVNNKMVVF-NENKTLFFKLSIVGTWP-SGTANRSMQLTFS-GSVPD 87
522-1                SSGT---LAPFFDTVNNKMVVF-NENKTLFFKLSIVGTWP-SGTANRSMQLTFS-GSVPD 87
2534-1               SSGT---LAPFFDTVNNKMVVF-NENKTLFFKLSIVGTWP-SGTANRSMQLTFS-GSVPD 87
FlexK-404            SSGT---LAPFFDTVNNKMVVF-NENKTLFFKLSIVGTWP-SGTANRSMQLTFS-GSVPD 87
Eco82-1              SSGT---LAPFFDTVNNKMVVF-NENKTLFFKLSIVGTWP-SGTANRSMQITFS-GSVPD 87
UMNK-1               SSGT---LAPFFDTVNNKMVVF-NENKTLFIKLSIVGTWP-SGTANRSMQLTFS-GSVPD 87
CUMT8                SSGT---LAPFFDTVNNKMVVF-NENKTLFFKLSIVGTWP-SGTANRSMQLTFS-GSVPD 87
2741-1               SSGT---LAPFFDTVNNKMVVF-NENKTLFFKLSIVGTWP-SGTTNRSMQLTFS-GSVPD 87
LB22-1               SSGT---LAPFFDTVNNKMVVF-NENKTLFFKLSIVGTWP-SGTANRSMQLTFS-GSVPD 87
2071-1               SSGT---LAPFFDTVNNKMVVF-NENKTLFFKLSIVGTWP-SGTANRSMQLTFS-GSVPD 87
3493-1               SSGT---LAPFFDTVNNKMVVF-NENKTLFFKLSIVGTWP-SGTANRSMQLTFS-GSVPD 87
7901-1               SSGT---LAPFFDTVNNKMVVF-NENKTLFFKLSIVGTWP-SGTANRSMQLTFS-GSVPD 87
8351-1               SSGT---LAPFFDTVNNKMVVF-NENKTLFFKLSIVGTWP-SGTANRSMQLTFS-GSVPD 87
C227-1               SSGT---LAPFFDTVNNKMVVF-NENKTLFFKLSIVGTWP-SGTANRSMQLTFS-GSVPD 87
C236-1               SSGT---LAPFFDTVNNKMVVF-NENKTLFFKLSIVGTWP-SGTANRSMQLTFS-GSVPD 87
9591-1               SSGT---LAPFFDTVNNKMVVF-NENKTLFFKLSIVGTWP-SGTANRSMQLTFS-GSVPD 87
4404-1               SSGT---LAPFFDTVNNKMVVF-NENKTLFFKLSIVGTWP-SGTANRSMQLTFS-GSVPD 87
4522-1               SSGT---LAPFFDTVNNKMVVF-NENKTLFFKLSIVGTWP-SGTANRSMQLTFS-GSVPD 87
3677-1               SSGT---LAPFFDTVNNKMVVF-NENKTLFFKLSIVGTWP-SGTANRSMQLTFS-GSVPD 87
4623-1               SSGT---LAPFFDTVNNKMVVF-NENKTLFFKLSIVGTWP-SGTANRSMQLTFS-GSVPD 87
C1-1                 SSGT---LAPFFDTVNNKMVVF-NENKTLFFKLSIVGTWP-SGTANRSMQLTFS-GSVPD 87
C2-1                 SSGT---LAPFFDTVNNKMVVF-NENKTLFFKLSIVGTWP-SGTANRSMQLTFS-GSVPD 87
C3-1                 SSGT---LAPFFDTVNNKMVVF-NENKTLFFKLSIVGTWP-SGTANRSMQLTFS-GSVPD 87
C4-1                 SSGT---LAPFFDTVNNKMVVF-NENKTLFFKLSIVGTWP-SGTANRSMQLTFS-GSVPD 87
C5-1                 SSGT---LAPFFDTVNNKMVVF-NENKTLFFKLSIVGTWP-SGTANRSMQLTFS-GSVPD 87
Dec12D               SSGT---LAPFFDTVNNKMVVF-NENKTLFFKLSIVGTWP-SGTANRSMQLTFS-GSVPD 87
Sf6                  SSGT---LAPFFDTVNNKMVVF-NENKTLFFKLSIVGTWP-SGTANRSMQLTFS-GSVPD 87*
EC4113               SSGT---LAPFFDTVNNKMVVF-NENKTLFFKLSIVGTWP-SGTANRSMQLTFS-GSVPD 87
STEC94C-1            SSGT---LAPFFDTVNNKMVVF-NENKTLFFKLSIVGTWP-SGTANRSMQLTFS-GSVPD 87
Ferg2                SSGT---LAPFFDTVNNKMVVF-NENKTLFFKLSIVGTWP-SGTANRSMQLTFS-GSVPD 87
TA271-1              SSGT---LAPFFDTVNNKMVVF-NENKTLFFKLSIVGTWP-SGTANRSMQLTFS-GSVPD 87
Eco989-1             SSGT---LAPFFDTVNNKMVVF-NENKTLFFKLSIVGTWP-SGTANRSMQLTFS-GSVPD 87
Ferg1                SSGT---LAPFFDTVNNKMVVF-NENKTLFFKLSIVGTWP-SGTANRSMQLTFS-GSVPD 87
HS1                  SSGT---LAPFFDTVNNKMVVF-NENKTLFFKLSIVGTWP-SGTANRSMQLTFS-GSVPD 87*
M863-1               SSGT---LAPFFDTVNNKMVVF-NENKTLFFKLSIVGTWP-SGTANRSMQLTFS-GSVPD 87
E1167-1              SSGT---LAPFFDTVNNKMVVF-NENKTLLFKLSIVGTWP-SGTANRSMQLTFS-GSVPD 87
Dec10C-1             SSGS---LAPFFDTVNNKMVVF-NENKTLFFKLSIVGTWP-SGTANRSMQLTFS-GSVPD 87
Dec10D-1             SSGS---LAPFFDTVNNKMVVF-NENKTLFFKLSIVGTWP-SGTANRSMQLTFS-GSVPD 87
Dec10B-1             SSGS---LAPFFDTVNNKMVVF-NENKTLFFKLSIVGTWP-SGTANRSMQLTFS-GSVPD 87
Dec9E-1              SSGS---LAPFFDTVNNKMVVF-NENKTLFFKLSIVGTWP-SGTANRSMQLTFS-GSVPD 87
Dec9D-1              SSGS---LAPFFDTVNNKMVVF-NENKTLFFKLSIVGTWP-SGTANRSMQLTFS-GSVPD 87
Dec9C-1              SSGS---LAPFFDTVNNKMVVF-NENKTLFFKLSIVGTWP-SGTANRSMQLTFS-GSVPD 87
Dec9B-1              SSGS---LAPFFDTVNNKMVVF-NENKTLFFKLSIVGTWP-SGTANRSMQLTFS-GSVPD 87
105-1                SSGS---LAPFFDTVNNKMVVF-NENKTLFFKLSIVGTWP-SGTANRSMQLTFS-GSVPD 87
Dec9A-1              SSGS---LAPFFDTVNNKMVVF-NENKTLFFKLSIVGTWP-SGTANRSMQLTFS-GSVPD 87
P18                  SSGS---LAPFFDTVNNKMVVF-NENKTLFFKLSIVGTWP-SGTANRSMQLTFS-GSVPD 87
30-1                 SSGS---LAPFFDTVNNKMVVF-NENKTLFFKLSIVGTWP-SGTANRSMQLTFS-GSVPD 87
9952                 SSGS---LAPFFDTVNNKMVVF-NENKTLFFKLSIVGTWP-SGTANRSMQLTFS-GSVPD 87
EC1865               SSGS---LAPFFDTVNNKMVVF-NENKTLFFKLSIVGTWP-SGTANRSMQLTFS-GSVPD 87
1629-1               SSGS---LAPFFDTVNNKMVVF-NENKTLFFKLSIVGTWP-SGTANRSMQLTFS-GSVPD 87
224-1                SSGS---LAPFFDTVNNKMVVF-NENKTLFFKLSIVGTWP-SGTANRSMQLTFS-GSVPD 87
26-1                 SSGS---LAPFFDTVNNKMVVF-NENKTLFFKLSIVGTWP-SGTANRSMQLTFS-GSVPD 87
21-1                 SSGS---LAPFFDTVNNKMVVF-NENKTLFFKLSIVGTWP-SGTANRSMQLTFS-GSVPD 87
9942-1               SSGS---LAPFFDTVNNKMVVF-NENKTLFFKLSIVGTWP-SGTANRSMQLTFS-GSVPD 87
Flex8401             SSGS---LTPFFDTVNNKMVVF-NENKTLFFKLSIVGTWP-SGTANRSMQLTFS-GAVPD 87
FlexM90T             SSGS---LTPFFDTVNNKMVVF-NENKTLFFKLSIVGTWP-SGTANRSMQLTFS-GAVPD 87
phage31              VYGS---WAPMFDIINDKMVAAKNDDRTLLYKIAITGTFD-NTSSTNALTLTTTIGSNID 232
44RR2-8torf183       VYGS---WAPMFDIINDKMVAAKNDDRTLLYKIAITGTFD-NTSSTNALTLTTTIGSNID 232
CMB120orf226         NPSSPTGLAPFFKLSDHKFHAF-PYDSILPVKVNIVGSWS-GSTSNRTMILDFV-GSVGN 95
phiHS19              NPSSPTGLAPFFKLSDHKFHAF-PYDSILPVKVNIVGSWS-GSTSNRTMILDFV-GSVGN 106
STML-13-1            NPSSPTGLAPFFKLSDHKFHAF-PYDSILPVKVNIVGSWS-GSTSNRTMILDFV-GSVGN 95
PhaxI                NPSSPTGLAPFFKLSDHKFHAF-PYDSILPVKVNIVGSWS-GSTSNRTMIVDFV-GSVGN 106
Vi01                 NPSSPTGLAPFFKLSDHKFHAF-PYDSILPVKVNIVGSWS-GSTSNRTMIVDFV-GSLGN 106
SFP10                NPSSPTGLAPFFKLSDHKFHAF-PYDSILPVKVNIVGSWS-GSTSNRTMILDFV-GSVGN 106
Limestone1           HPASVTGLAPFFKLSDHKFHAF-PVDSILPVKVNIIGTWS-GSTSNRTMLLDFV-GSTGN 106
AeromonasSUU         AFGT---LAPFFNTVSNKLNAY-NDNASLPFKLNLAGTWS-AGTSNRSLQLDFV-GTNGN 101
RB43                 TTGT---LEPFFKITDNKLHPF-NENSTLTFKLNLKGTFTGATTAQRSVTLDFV-GTQGN 173
Vibrio0395-1174      IFGS---LLPFFDTAANLLRSF-NDDASLHFKANFIGSFP-GSAATRSLELDFL-GTEGN 102
Vibrio0395-1257      IFGS---LLPFFDTAANLLRSF-NDDASLHFKANFIGSFP-GSAATRSLELDFL-GTEGN 102
VibrioRC27           IFGS---LLPFFDTAANLLRSF-NDDASLHFKANFIGSFP-GSAATRSLELDFL-GTEGN 102
VibrioTMA21          IFGS---LLPFFDTVANLLRSF-NDDASLHFKANFIGSFP-GSAATRSLELDFL-GTEGN 102
VibrioTM11079        VFGS---LLPFFDTAANLLRSF-NDDASLHFKANFIGSFP-GSAATRSLELDFL-GTEGN 102
VibrioTM2740         VFGS---LLPFFDTAANLLRSF-NDDASLHFKANFIGSFP-GSAATRSLELDFL-GTEGN 102
Vibrio12129          VFGN---LLPFFDTAANLLRSF-NDDASLHFKANFIGSFP-GSAATRSLELDFL-GTEGN 102
VirbiroSX-4          VFGS---LLPFFDTANNLLRAF-NDNRSLYFKANFTGTFP-GSAAIRSLELDFA-GTQGN 102
                       ..     *:*.   . :      :  *  *  : *::    :: .:: :    *:  :
```

```
Flex2017B               TLVSSRNAATTT-DNILLATFFSVDKDGFLATNGSTLTIQSNGAAFTATTIKIIAEQ--- 143
Flex2457                TLVSSRNAATTT-DNILLATFFSVDKDGFLATNGSTLTIQSNGAAFTATTIKIIAEQ--- 143
Flex301                 TLVSSRNAATTT-DNILLATFFSVDKDGFLATNGSTLTIQSNGAAFTATTIKIIAEQ--- 143
FlexK-304               TLVSSRNAATTT-DNILLATFFSVDKDGFLATNGSTLTIQSNGAAFTATTIKIIAEQ--- 143
FlexK-671               TLVSSRNAATTT-DNILLATFFSVDKDGFLATNGSTLTIQSNGAAFTATTIKIIAEQ--- 143
Flex2930-71             TLVSSRNAATTT-DNILLATFFSVDKDGFLATNGSTLTIQSNGAAFTATTIKIIAEQ--- 143
Flex2747-71             TLVSSRNAATTT-DNILLATFFSVDKDGFLATNGSTLTIQSNGAAFTATTIKIIAEQ--- 143
E1520-1                 TLVSSRNAATTT-DNILLATFFSVDKDGFLATNGSTLTIQSNGAAFTATTIKIIAEQ--- 143
B354-1                  TLVSSRNAATTT-DNILLATFFSVDKDGFLATNGSTLTIQSNGAAFTATTIKIIAEQ--- 143
B7A1                    TLVSSRNAATTT-DNILLATFFSVDKDGFLATNGSTLTIQSNGAAFTATTIKIIAEQ--- 143
Boyd-1                  TLVSSRNAATTT-DNILLATFFSVDKDGFLVTNGSTLTIQSNGAAFTATTIKIIAEQ--- 143
Ferg3                   TLVSSRNSATTT-DNILLATFFSVDKDGFLATNGSTLTIQSNGAAFTATIIKIIAEQ--- 143
Flex2017                TLVSSRNAATTT-DNILLATFFSVDKDGFLATNGSTLTIQSNGAAFTATIIKIIAEQ--- 143
9570-1                  TLVSSRNSATTT-DNILLATFFSVDKDGFLATNGSTLTIQSNGAAFTATTIKIIAEQ--- 143
Dec12B                  TLVSSRNAATTT-DNILLATFFSVDKDGFLASNGSTLTIQSNGAAFTATTIKIIAEQ--- 143
9574-1                  TLVSSRNAATTT-DNILLATFFSVDKDGFLATNGSTLTIQSNGAAFTATTIKIIAEQ--- 143
W-1                     TLVSSRNAATTT-DNILLATFFSVDKDGFLATNGSTLTIQSNGAAFTATTIKIIAEQ--- 143
K011-1                  TLVSSRNAATTT-DNILLATFFSVDKDGFLATNGSTLTIQSNGAAFTATTIKIIAEQ--- 143
H494-1                  TLVSSRNAATTT-DNILLATFFSVDKDGFLATNGSTLTIQSNGAAFTATTIKIIAEQ--- 143
1632-1                  TLVSSRNAATTT-DNILLATFFSVDKDGFLATNGSTLTIQSNGAAFTATTIKIIAEQ--- 143
B088-1                  TLVSSRNAATTT-DNILLATFFSVDKDGFLATNGSTLTIQSNGAAFTATTIKIIAEQ--- 143
522-1                   TLVSSRNAATTT-DNILLATFFSVDKDGFLATNGSTLTIQSNGAAFTATTIKIIAEQ--- 143
2534-1                  TLVSSRNAATTT-DNILLATFFSVDKDGFLATNGSTLTIQSNGAAFTATTIKIIAEQ--- 143
FlexK-404               TLVSSRNAATTT-DNILLATFFSVDKDGFLATNGSTLTIQSNGAAFTATTIKIIAEQ--- 143
Eco82-1                 TLVSSRNAATTT-DNILLATFFSVDKDGFLATNGSTLTIQSNGAAFTATTIKIIAEQ--- 143
UMNK-1                  TLVSSRNAATTT-DNILLATFFSVDKDGFLATNGSTLTIQSNGAAFTATTIKIIAEQ--- 143
CUMT8                   TLVSSRNAATTT-DNILLATFFSVDKDGFLATNGSTLTIQSNGAAFTATTIKIIAEQ--- 143
2741-1                  TLVSSRNAATTT-DNILLATFFSVDKDGFLATNGSTLTIQSNGAAFTATTIKIIAEQ--- 143
LB22-1                  TLVSSRNAVTTT-DNILLATFFSVDKDGFLATNGSTLTIQSNGAAFTATTIKIIAEQ--- 143
2071-1                  TLVSSRNAVTTT-DNILLATFFSVDKDGFLATNGSTLTIQSNGAAFTATTIKIIAEQ--- 143
3493-1                  TLVSSRNAVTTT-DNILLATFFSVDKDGFLATNGSTLTIQSNGAAFTATTIKIIAEQ--- 143
7901-1                  TLVSSRNAVTTT-DNILLATFFSVDKDGFLATNGSTLTIQSNGAAFTATTIKIIAEQ--- 143
8351-1                  TLVSSRNAVTTT-DNILLATFFSVDKDGFLATNGSTLTIQSNGAAFTATTIKIIAEQ--- 143
C227-1                  TLVSSRNAVTTT-DNILLATFFSVDKDGFLATNGSTLTIQSNGAAFTATTIKIIAEQ--- 143
C236-1                  TLVSSRNAVTTT-DNILLATFFSVDKDGFLATNGSTLTIQSNGAAFTATTIKIIAEQ--- 143
9591-1                  TLVSSRNAVTTT-DNILLATFFSVDKDGFLATNGSTLTIQSNGAAFTATTIKIIAEQ--- 143
4404-1                  TLVSSRNAVTTT-DNILLATFFSVDKDGFLATNGSTLTIQSNGAAFTATTIKIIAEQ--- 143
4522-1                  TLVSSRNAVTTT-DNILLATFFSVDKDGFLATNGSTLTIQSNGAAFTATTIKIIAEQ--- 143
3677-1                  TLVSSRNAVTTT-DNILLATFFSVDKDGFLATNGSTLTIQSNGAAFTATTIKIIAEQ--- 143
4623-1                  TLVSSRNAVTTT-DNILLATFFSVDKDGFLATNGSTLTIQSNGAAFTATTIKIIAEQ--- 143
C1-1                    TLVSSRNAVTTT-DNILLATFFSVDKDGFLATNGSTLTIQSNGAAFTATTIKIIAEQ--- 143
C2-1                    TLVSSRNAVTTT-DNILLATFFSVDKDGFLATNGSTLTIQSNGAAFTATTIKIIAEQ--- 143
C3-1                    TLVSSRNAVTTT-DNILLATFFSVDKDGFLATNGSTLTIQSNGAAFTATTIKIIAEQ--- 143
C4-1                    TLVSSRNAVTTT-DNILLATFFSVDKDGFLATNGSTLTIQSNGAAFTATTIKIIAEQ--- 143
C5-1                    TLVSSRNAVTTT-DNILLATFFSVDKDGFLATNGSTLTIQSNGAAFTATTIKIIAEQ--- 143
Dec12D                  TLVSSRNAATTT-DNILLATFFSVDKDGFLATNGSTLTIQSNGAPFTATTIKIIAEQ--- 143
Sf6                     TLVSSRNSATTT-DNILLATFFSVDKDGFLATNGSTLTIQSNGASFTATTIKIIAEQ--- 143*
EC4113                  TLVSSRNSATTT-DNILLATFFSVDKDGFLATNGSTLTIQSNGAAFTATTIKIIAEQ--- 143
STEC94C-1               TLVSSRNSATTT-DNILLATFFSVDKDGFLATNGSTLTIQSNGAAFTATTIKIIAEQ--- 143
Ferg2                   TLVSSRNSATTT-DNILLATFFSVDKDGFLATNGSTLTIQSNGAAFTATTIKIIAEQ--- 143
TA271-1                 TLVSSRNSATTT-DNILLATFFSVDKDGFLATNGSTLTIQSNGAAFTATTIKIIAEQ--- 143
Eco989-1                TLVSSRNSATTT-DNILLATFFSVDKDGFLATNGSTLTIQSNGAAFTATTIKIIAEQ--- 143
Ferg1                   TLVSSRNSATTT-DNILLATFFSVDKDGFLATNGSTLTIQSNGAAFTATTIKIIAEQ--- 143
HS1                     TLVSSRNSATTT-DNILLATFFSVDKDGFLATNGSTLTIQSNGAAFTATTIKIIAEQ--- 143*
M863-1                  TLVSSRNSATTT-DNILLATFFSVDKDGFLATNGSALTIQSNGAAFTATTIKIIAEQ--- 143
E1167-1                 TLVSSRNSATTT-DNILLATFFSVDKDGFLATNGSTLTIQSNGAAFTATTIKIIAEQ--- 143
Dec10C-1                TLVSSRNSATTT-DNILLATFFSVDKDGFLATNGSTLTIQSNGAAFTATTIKIIAEQ--- 143
Dec10D-1                TLVSSRNSATTT-DNILLATFFSVDKDGFLATNGSTLTIQSNGAAFTATTIKIIAEQ--- 143
Dec10B-1                TLVSSRNSATTT-DNILLATFFSVDKDGFLATNGSTLTIQSNGAAFTATTIKIIAEQ--- 143
Dec9E-1                 TLVSSRNSATTT-DNILLATFFSVDKDGFLATNGSTLTIQSNGAAFTATTIKIIAEQ--- 143
Dec9D-1                 TLVSSRNSATTT-DNILLATFFSVDKDGFLATNGSTLTIQSNGAAFTATTIKIIAEQ--- 143
Dec9C-1                 TLVSSRNSATTT-DNILLATFFSVDKDGFLATNGSTLTIQSNGAAFTATTIKIIAEQ--- 143
Dec9B-1                 TLVSSRNSATTT-DNILLATFFSVDKDGFLATNGSTLTIQSNGAAFTATTIKIIAEQ--- 143
105-1                   TLVSSRNSATTT-DNILLATFFSVDKDGFLATNGSTLTIQSNGAAFTATTIKIIAEQ--- 143
Dec9A-1                 TLVSSRNSATTT-DNILLATFFSVDKDGFLATNGSTLTIQSNGAAFTATTIKIIAEQ--- 143
P18                     TLVSSRNSATTT-DNILLATFFSVDKDGFLATNGSTLTIQSNGAAFTATTIKIIAEQ--- 143
30-1                    TLVSSRNSATTT-DNILLATFFSVDKDGFLATNGSTLTIQSNGAAFTATTIKIIAEQ--- 143
9952                    TLVSSRNSATTT-DNILLATFFSVDKDGFLATNGSTLTIQSNGAAFTATTIKIIAEQ--- 143
EC1865                  TLVSSRNSATTT-DNILLATFFSVDKDGFLATNGSTLTIQSNGAAFTATTIKIIAEQ--- 143
1629-1                  TLVSSRNSATTT-DNILLATFFSVDKDGFLATNGSTLTIQSNGAAFTATTIKIIAEQ--- 143
224-1                   TLVSSRNSATTT-DNILLATFFSVDKDGFLATNGSTLTIQSNGAAFTATTIKIIAEQ--- 143
26-1                    TLVSSRNSATTT-DNILLATFFSVDKDGFLATNGSTLTIQSNGAAFTATTIKIIAEQ--- 143
21-1                    TLVSSRNSATTT-DNILLATFFSVDKDGFLATNGSTLTIQSNGAAFTATTIKIIAEQ--- 143
9942-1                  TLVSSRNSATTT-DNILLATFFSVDKDGFLATNGSTLTIQSNGAAFTATTIKIIAEQ--- 143
Flex8401                TLVSSRNSATTT-DNILLATFFSVDKDGFLAANGSTLTIQSNGAAFTATTIKIIAEQ--- 143
FlexM90T                TLVSSRNSATTT-DNILLATFFSVDKDGFLAANGSTLTIQSNGAAFTATTIKIIAEQ--- 143
phage31                 VSSAVRLPNQNP-QNINYISLISVDKNGNFATNGASLTLRSTTSDFTITRVRLIAEQ--- 288
44RR2-8torf183          VSSAVRLPNQNP-QNINYISLISVDKNGNFATNGASLTLRSTTSDFTITRVRLIAEQ--- 288
CMB120orf226            QLSRSRDASVPPPDTLSFITFFSVDKDGNLATNGAQMKLYSYGGDFTITEVVLIAEQVVP 155
phiHS19                 QLSRSRDASVPPPDTLSFITFFSVDKDGNLATNGAQMKLYSYGGDFTITEVVLIAEQVVP 166
STML-13-1               QLSRSRDASVPPPDTLSFITFFSVDKDGNLATNGAQMKLYSYGGDFTITEVVLIAEQVVP 155
PhaxI                   QLSRSRDASVPPPDTLSFITFFSVDKDGNLATNGAQIKLYSYGGDFTITEVVLIAEQVVP 166
Vi01                    QLSRSRDASVPPPDTLSFITFFSVDKDGNLATNGAQIKLYSYGGDFTITEVVLIAEQVVP 166
SFP10                   QLSRSRDASVPPPDTLSFITFFSVDKDGNLATNGAQMKLYSYGGDFTITEVVLIAEQVVP 166
Limestone1              QLSKSRDSSLPPPDVLSFITFFSVDKAGNLVTNGAQMKLNSYGGDFTINEIILIAEQVVP 166
AeromonasSUU            RLVASRDAAVTT-DVITLATFFSIDRDGGIVTSGTSPIIRSNGGSYTLNSVLLIAEQATR 160
RB43                    TLTLNRSQSVTT-STLSFVTFFSVDKNGNMVTNGTAFNIFANGSPFTVSEVLLVAEQRTT 232
Vibrio0395-1174         RLVQNRSLEVDE-DVMTFSTFFSVDKNGNIATNGTAIQIRANGRDFTCTKLLLIAEQETY 161
Vibrio0395-1257         RLVQNRSLEVDE-DVMTFSTFFSVDKNGNIATNGTAIQIRANGRDFTCTKLLLIAEQETY 161
VibrioRC27              RLVQNRSLEVDE-DVMTFSTFFSVDKNGNIATNGTAIQIRANGRDFTCTKLLLIAEQETY 161
VibrioTMA21             RLVQNRSLEVDE-DVMTFSTFFSVDKNGNIATNGTAIQIRANGRDFTCTKLLLIAEQETY 161
VibrioTM11079           RLVQNRSLEVDE-DVMTFSTFFSVDKNGNIATNGTAIQIRANGRDFTCTKLLLIAEQETY 161
VibrioTM2740            RLVQNRSLEVDE-DVMTFSTFFSVDKNGNIATNGTAIQIRANGRDFTCTKLLLIAEQETY 161
Vibrio12129             RLVQNRSLEVDE-DVMTFSTFFSVDKNGNIATNGTAIQIRANGRDFTCTKLLLIAEQETY 161
VirbiroSX-4             RLVQNRSLEVAE-DVMNFSTFFSVDKNGNIATNGTAIQIRANGLDFTCTKLLLIAEQETY 161
                             *       . :   :::*:*: * :.:.*:   : :    :* . : ::***
                            P P               . .  .     .            .       ...
```

```
Flex2017B          ---------
Flex2457           ---------
Flex301            ---------
FlexK-304          ---------
FlexK-671          ---------
Flex2930-71        ---------
Flex2747-71        ---------
E1520-1            ---------
B354-1             ---------
B7A1               ---------
Boyd-1             ---------
Ferg3              ---------
Flex2017           ---------
9570-1             ---------
Dec12B             ---------
9574-1             ---------
W-1                ---------
K011-1             ---------
H494-1             ---------
1632-1             ---------
B088-1             ---------
522-1              ---------
2534-1             ---------
FlexK-404          ---------
Eco82-1            ---------
UMNK-1             ---------
CUMT8              ---------
2741-1             ---------
LB22-1             ---------
2071-1             ---------
3493-1             ---------
7901-1             ---------
8351-1             ---------
C227-1             ---------
C236-1             ---------
9591-1             ---------
4404-1             ---------
4522-1             ---------
3677-1             ---------
4623-1             ---------
C1-1               ---------
C2-1               ---------
C3-1               ---------
C4-1               ---------
C5-1               ---------
Dec12D             ---------
Sf6                ---------*
EC4113             ---------
STEC94C-1          ---------
Ferg2              ---------
TA271-1            ---------
Eco989-1           ---------
Ferg1              ---------
HS1                ---------*
M863-1             ---------
E1167-1            ---------
Dec10C-1           ---------
Dec10D-1           ---------
Dec10B-1           ---------
Dec9E-1            ---------
Dec9D-1            ---------
Dec9C-1            ---------
Dec9B-1            ---------
105-1              ---------
Dec9A-1            ---------
P18                ---------
30-1               ---------
9952               ---------
EC1865             ---------
1629-1             ---------
224-1              ---------
26-1               ---------
21-1               ---------
9942-1             ---------
Flex8401           ---------
FlexM90T           ---------
phage31            ---------
44RR2-8torf183     ---------
CMB120orf226       LYMTSI--- 161
phiHS19            LYMTSI--- 172
STML-13-1          LYMTSI--- 161
PhaxI              LYMTSI--- 172
Vi01               LYMTSI--- 172
SFP10              LYMSSI--- 172
Limestone1         LYMNTI--- 172
AeromonasSUU       QTQISAV-- 167
RB43               Q-------- 233
VibrioO395-1174    STEILGGGA 170
VibrioO395-1257    STEILGGGA 170
VibrioRC27         STEILGGGA 170
VibrioTMA21        STEMLGGGA 170
VibrioTM11079      STEMLGGGA 170
VibrioTM2740       STEMLGGGA 170
Vibrio12129        STEILGGGA 170
VirbiroSX-4        STEMLGGGA 170
```

Figure S3. Conserved residues in Sf6 tail needle knob-like proteins.

The proteins shown in the figure S1 tree were aligned by Clustal W at the web site http://www.ebi.ac.uk/Tools/msa/clustalw2/ [Larkin *et al.* (2007) Clustal W and Clustal X version 2.0. Bioinformatics 23: 2947-2948; asjens SR, Thuman-Commike PA (2011) Evolution of mosaically related tailed bacteriophage genomes seen through the lens of phage P22 virion assembly. Virology 411: 393-415]. The tail needle knob sequences containing only residues homologous to Sf6 amino acids 140 to 282 from P22-like phages are shown above with labels on the left in red text (Sf6 and HS1 are noted with an asterisk (*) at the right end of those lines. The complete sequences of the extant homologues of this domain from outside the P22-like phage group are aligned below with labels in black text of the left. The consensus sequence is shown below with asterisks (*) marking universally conserved amino acids, colons (:) marking amino acids with strongly similar properties - scoring > 0.5 in the Gonnet PAM 250 matrix, and periods (.) marking amino acids with weakly similar properties - scoring ≤ 0.5 in the Gonnet PAM 250 matrix. In the bottom line red boxes indicate the universal conservation of the residues that contact the bound glutamate, green boxes indicate other universally conserved amino acids, and "P" marks amino acids that contact the phosphate ion on the 3-fold axis in Sf6.
