# Supplementary material for: The Tip of the Tail Needle Affects the Rate of DNA Delivery by Bacteriophage P22
Source: PLoS One. 2013 Aug 12;8(8):e70936. doi: 10.1371/journal.pone.0070936 (PMC3741392; doi:10.1371/journal.pone.0070936)
Supplement: Figure S4 — The hybrid P22:HS1-1 hybrid needle is incorporated into the virion. (PDF) [file pone.0070936.s004.pdf]

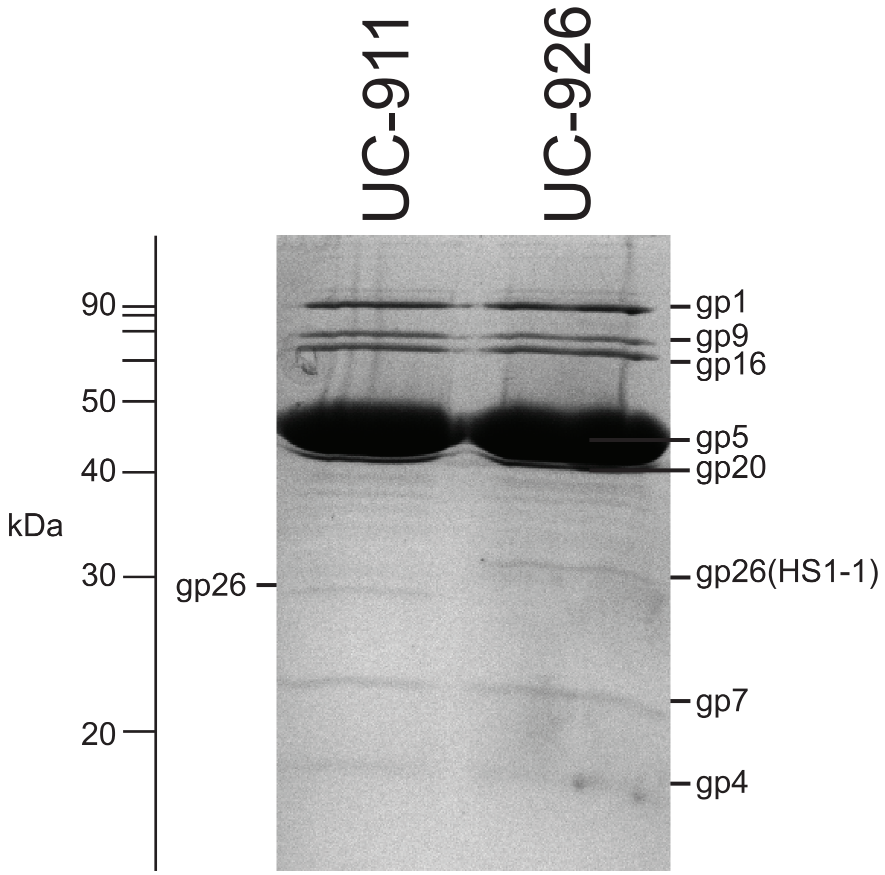

Figure S4. The hybrid P22:HS1-1 hybrid needle is incorporated into the virion.

Virions of P22 UC-911 and P22 UC-926 were purified through two successive CsCl step gradient centrifugations [Earnshaw W, Casjens S, Harrison S (1976) Assembly of the head of bacteriophage P22, X-ray diffraction from heads, proheads and related structures. J Mol Biol 104: 387-410], their proteins were separated in a 12% polyacrylamide SDS electrophoresis gel, and the gel was stained with Coomasie Brilliant Blue. The two virions have identical structural proteins except there is no band in UC-926 that corresponds to the P22 needle protein (gp26); however, there is a band of similar intensity that migrates somewhat slower UC-926 in virions. The P22:HS1-1 needle is expected to be about 6 kDa larger than the P22 protein, and we therefore suggest that this UC-926 protein band is the hybrid gp26. We note that several of the P22 virion proteins do not migrate precisely at the positions predicted by their molecular weights, and the kDa scale at the left was generated from commercially available size standards (BioRad).
