# Supplementary material for: The Tip of the Tail Needle Affects the Rate of DNA Delivery by Bacteriophage P22
Source: PLoS One. 2013 Aug 12;8(8):e70936. doi: 10.1371/journal.pone.0070936 (PMC3741392; doi:10.1371/journal.pone.0070936)
Supplement: Table S1 — Oligonucleotides used in this study. (PDF) [file pone.0070936.s005.pdf]

Table S1
Oligonucleotides used in this study

Oligonucleotide[a]	Sequence (5'to 3')
A	ACGAAGCTGGACAGGGTGCTTATGACGCACAGGTAAAAAATGATGTTAAGA<br>CCCACTTTCACATT
B	ACCATTTCTATCAGGTCGATGTTGCGTGTTGGAGTGAATGTAATCCTAAGC<br>ACTTGTCTCCTG
C	GGACAGGGTGCTTATGACGCACAGGTAAAAAATGATGAGCAGGATGTGATT<br>CTCGCTGACCAT
D	CATTTCTATCAGGTCGATGTTGCGTGTTGGAGTGAATGTAATCATTTACTG<br>CTCCGCGATTATC
E	GGGTTACGACTGCCGAGAACAATATTTCGGCATTGCAGGCTGACTACGTAC<br>CTGTTGACAATTAATCATCCGCA
F	CAACCATTTCTATCAGGTCGATGTTGCGTGTTGGAGTGAATGTAATCATTA<br>TCAGCACTGTCCTGCTCCTT
G	GACTGCCGAGAACAATATTTCGGCATTGCAGGCTGACTACGCCACCACTCG<br>CAAGAAATCA
H	CTATCAGGTCGATGTTGCGTGTTGGAGTGAATGTAATCATTACTGCTCCGC<br>GATTATCT
I	CATCGATGCTCTGGAGTATGCAACCACACGCAAGAAGTCATTAAGACCCAC<br>TTTCACATT
J	GCGCTGTCGGGATGGTTACAGATACACCAGAGTAAACAACCTAAGCACTTG<br>TCTCCTG
K	GTGTTCAACGAGAACAAAACCCTGTTCTTCTTAAGACCCACTTTCACATT
L	CACTGGGCCACGTCCCGACAATCGACAGCTAAGCACTTGTCTCCTG
M	GGCGACAACGACCGATAACATCCTGTTAGCTACGTTCTTCTAAGACCCAC<br>TTTCACATT
N	TGGTTAACGTGCTGCCATTTGTGGCAAGAAAGCCGTCTTTCTAAGCACTT<br>GTCTCCTG
O	CCAACGAGGCCGGACAGGGCGCTTATGATGCACAGGTC
P	GCTCCGCGATTATCTTGATGGTTGTGGCAGTAAACGACGC
Q	ACGAAGCTGGACAGGGTGCTTATGACGCACAGGTAAAAAATGATGCCTGTT<br>GACAATTA
R	ACCATTTCTATCAGGTCGATGTTGCGTGTTGGAGTGAATGTAATCTCAGCA<br>CTGTCCTGCTCCTT
S	GGCAGACCCGTCACTTAATAATCC
T	TTGCGTGTTGGAGTGAATGTAATCATTAATCAATCAACCCATGTGCTTATA<br>AAAAGGTAGAAAGCAATACC

a. Oligonucleotides were synthesized by the University of Utah oligonucleotide core facility.
